# TGF-β2 drives lipid droplet accumulation in chondrocytes through the TβRI/p-smad3/fabp5 axis

**DOI:** 10.1016/j.jbc.2026.113317

**Published:** 2026-07-06

**Authors:** Jieya Wei, Caixia Pi, Yu Qi, Wenbin Yang, Jianxun Sun, Xin Xu, Jing Xie

**Affiliations:** State Key Laboratory of Oral Diseases & National Center for Stomatology & National Clinical Research Center for Oral Diseases, West China Hospital of Stomatology, Sichuan University, Chengdu, Sichuan, China

**Keywords:** chondrocyte, Fabp5, lipid metabolism, osteoarthritis, receptor TβRI, transforming growth factor-β2, p-Smad3

## Abstract

Chondrocytes preserve cartilage lipid homeostasis by storing neutral lipids in lipid droplets and controlling their turnover through coordinated biochemical signaling. TGF-β2 is elevated in osteoarthritis and regulates key chondrocyte functions, including proliferation, differentiation, and cell death. However, whether and how TGF-β2 regulates chondrocyte lipid metabolism remains unknown. Here, we characterize TGF-β2-regulated lipid droplet accumulation and delineate the underlying mechanism. TGF-β2 drives neutral lipid and lipid droplet accumulation in chondrocytes and cartilage through upregulation of Fabp5. This effect requires TGF-β2 signaling through receptor TβRI, which induces the phosphorylation and nuclear translocation of Smad3, thereby promoting Fabp5 transcription. In this process, TGF-β2 induces enrichment of cellular lipid intermediates involved in lipid droplet accumulation. Collectively, our findings reveal a novel TGF-β2/TβRI/p-Smad3/Fabp5 signaling axis that regulates lipid storage in chondrocytes and suggest potential metabolic targets for maintaining cartilage homeostasis and treating related diseases.

Articular cartilage, lining the ends of opposing bones within synovial joints, is a connective tissue characterized by thinness, smoothness, and viscoelastic properties. This tissue features a high extracellular matrix content (up to 95% by volume) and a sparse chondrocyte population (1). It is essential for load distribution, shock absorption, friction minimization, and joint stability (2, 3). However, its intrinsic regenerative capacity is still a clinical challenge due to the absence of microvessels, lymphatic vessels, and neural networks, and this deficiency often leads to the expansion of articular cartilage damage into surrounding tissues such as the peripheral synovium and subchondral bone, ultimately initiating an irreversible cascade of symptoms including joint pain, stiffness, and functional impairment (4). Cartilage injury and subsequent irreversible degeneration are the main hallmark events of osteoarthritis, which is considered to be a type of lipid metabolism disorder with symptoms similar to systemic metabolic syndrome (5). Interestingly, a notable structural and metabolic feature of articular cartilage is its capacity to act as a lipid reservoir. Cartilage internalizes lipids diffusing from adjacent tissues and stores them either within the extracellular matrix or as cytoplasmic lipid droplets in chondrocytes, providing a readily available energy substrate for cellular metabolism (6, 7).

Lipid droplets are assembled around a core of neutral lipids, predominantly triacylglycerols and cholesterol esters, and are bounded by a phospholipid monolayer that is studded with specific surface proteins (8). They are first synthesized in the endoplasmic reticulum through the aggregation and budding of neutral lipids (9). During their maturation, tiny lipid droplets uptake lipids from the surface of endoplasmic reticulum, coalesce with smaller lipid particles, and fuse into larger ones (10). Serving as dynamic storage and transport systems for lipids, lipid droplets tightly participate in lipid metabolism, including lipolysis, lipophagy and lipogenesis, in response to cellular metabolic and energetic demands (11). Through the dynamic inter-organellar contact with the endoplasmic reticulum, mitochondria, Golgi apparatus, peroxisomes, and lysosomes, lipid droplets also serve as central hubs for organelle assembly, membrane synthesis/trafficking, ion signaling, protein degradation, and autophagy (12). Additionally, lipid droplets are involved in defense against lipotoxicity, regulation of oxidative stress, intervention in inflammatory and immune responses, and even shaping of the genomic landscape (13). In cartilage, previous evidence has revealed that excessive lipid droplet accumulation in chondrocytes can contribute to structural and functional damage to cartilage layers and trigger osteochondral diseases such as osteoarthritis (14, 15). Research from Park *et al*. showed that acetyl-CoA thioesterase 12 (Acot12) knockout-driven lipid droplet accumulation increased metabolic stress, impaired chondrocyte cellularity, and exacerbated cartilage degradation in osteoarthritis (16). In contrast, Lee *et al*. reported that lipid droplet accumulation mediated by protein kinase casein kinase 2 (Pkck2), six-transmembrane protein of prostate 2 (Stamp2), and fat-specific protein 27 (Fsp27) protected chondrocytes from lipotoxicity and represented a potential target for osteoarthritis interventions (17). However, the mechanism of chondrocyte lipid droplet formation and its importance in disease occurrence is still insufficiently understood.

Lipid droplet-associated protein regulators orchestrate the dynamic life cycle of lipid droplets through controlling lipid droplet formation, enlargement, merging, breakdown, and inter-organellar communication (18). These proteins can be categorized into multiple functional groups, including enzymes involved in lipid metabolism, membrane transporters facilitating organelle interactions, histones linking lipid droplets to nucleic acids, ribosomal proteins engaged in prokaryotic protein synthesis, protein degradation-associated proteins, and other signaling molecules (19). In this context, the fatty acid-binding protein (Fabp) family is considered to play an important role in the formation and function of lipid droplets. Fabps are highly conserved and widely expressed lipid chaperones with molecular weights ranging from 14 to 15 kDa (20). Characterized by a distinctive hydrophobic pocket, Fabps enable the reversible binding of fatty acids (21), which allows them to facilitate lipid metabolic compartmentalization by directing lipids to specific organelles, thereby regulating storage in lipid droplets, membrane biogenesis in the endoplasmic reticulum, oxidative breakdown in mitochondria and peroxisomes, and transcriptional control within the nucleus (22).

Reports have indicated that multiple biochemical factors, including inflammatory factors, chemokine factors and growth factors, can regulate lipid droplet accumulation (23, 24). The transforming growth factor-β (TGF-β) subfamily is a well-established regulator of cellular metabolism and exerts specific control over lipid regulatory pathways. For example, TGF-β1 is shown to have a protective role against microglia-mediated neuroinflammation *via* the suppression of cellular lipid accumulation (25). TGF-β2, induced by acidosis in tumor microenvironment, supports anoikis resistance and cancer cell invasiveness by Smad2 acetylation-induced fatty acid metabolism (26). TGF-β3 promotes trophoblast development in vertebrate embryos through acetyl-CoA synthetase short-chain family member 2 (ACSS2)-dependent lipid metabolism (27). In cartilage, TGF-β2 possesses distinct characteristics compared to TGF-β1 and β3. Its abundance increases significantly in deeper cartilage layers and across more chondrocytes as osteoarthritis progresses (28, 29). It exhibits distinct transcriptional regulatory properties, attributed to its promoter region, which harbors two TATA boxes, cAMP response elements, and a non-functional AP-1 binding site (30). While TGF-β1 and TGF-β3 transcript levels remain relatively stable, TGF-β2 transcripts show a sharp 5-fold decline in late-passage chondrocytes within high-density pellets, compromising their cartilage-forming capacity (31). Additionally, TGF-β2 precursors lack the Arg-Gly-Asp motif, which prevents their activation by integrin αvβ6 but enables activation by matrix metalloproteinase 2 and 3, and thrombospondin 1 (29). Structurally, the TGF-β2 monomer possesses an additional disulfide bond tethering its α-helix and potentially conferring functional distinctions in transport, activity, stability, and protein interactions (32). Moreover, the unique transforming growth factor-β receptor (TβR) binding profile of TGF-β2 modulates the stoichiometry and dynamics of initial receptor engagement, which ultimately defines the signaling threshold and spatiotemporal specificity for the activation of its indispensable transducer, TβRI (33). Functionally, TGF-β2 is a critical regulator of diverse chondrocyte activities, including proliferation, differentiation, migration, intercellular communication, and metabolic balance, thereby influencing cartilage development, homeostasis, and pathological states (29, 34).

Despite these unique properties and functional significance of TGF-β2, its role in chondrocyte lipid metabolism remains undefined. This study aims to investigate the importance of TGF-β2 in lipid accumulation and lipid droplet formation in chondrocytes to establish a molecular basis for chondrocyte lipid homeostasis and provide cues for preventive and therapeutic strategies for lipid metabolism-related osteochondral disorders.

## Results

### TGF-β2 drives lipid droplet accumulation

**Figure 1:**
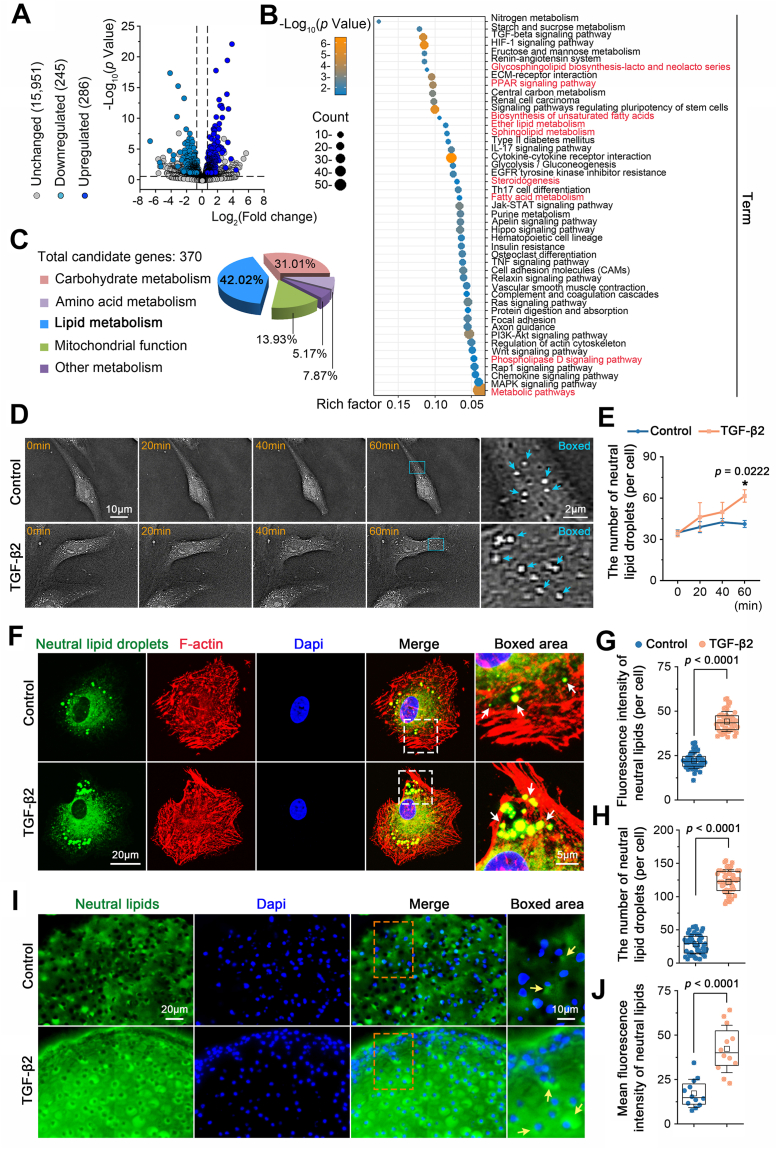
**TGF-β2 drives lipid droplet accumulation.***A*, Volcano plot based on bulk RNA sequencing showing that treatment with TGF-β2 at 5 ng/ml for 24 h upregulated 286 genes and downregulated 245 genes in chondrocytes. Fold change > 1.5 & *p* < 0.05. *B*, bubble chart based on KEGG analysis demonstrating the significant differences in lipid metabolism-related pathways (*red*) in chondrocytes induced by TGF-β2 at 5 ng/ml for 24 h. *C*, Pie chart showing that lipid metabolism-related genes constituted the largest metabolic category (42.02%) in chondrocytes induced by TGF-β2 at 5 ng/ml for 24 h. *D*, Label-free real-time cell monitoring system recording the dynamics of TGF-β2-induced lipid droplets in chondrocytes (n = 3). Scale bars: 10 μm (first four columns) and 2 μm (last column, enlarged images). *E*, line graph showing changes in average dynamic number of neutral lipid droplets in living chondrocytes induced by TGF-β2 at 5 ng/ml in (d). Data from 3 replicates of three independent experiments (n = 3) were analyzed by two-way repeated-measures ANOVA and are presented as mean ± SD. *F*, representative immunofluorescence micrographs showing increased neutral lipids and lipid droplets in chondrocytes after a 48-h treatment with 5 ng/ml TGF-β2. BODIPY 493/503 visualizes neutral lipids and lipid droplets (*green*), TRITC-phalloidin delineates F-actin cytoskeleton (*red*), and Dapi labels nuclei (*blue*). Images are representative of six independent experiments (n = 6). Scale bars: 20 μm (first four columns) and 5 μm (last column, enlarged images). *G*, mean fluorescence intensity quantification per chondrocyte validating the observed neutral lipid abundance in (*F*). Data from 50 individual cell replicates of six independent experiments (n = 6) were analyzed by unpaired two-tailed Student's *t* test and are presented as mean ± SD. *H*, quantitative analysis of average lipid droplet number per chondrocyte confirming the observed lipid droplet accumulation in (*F*). Data from 50 individual cell replicates of six independent experiments (n = 6) were analyzed by unpaired two-tailed Student's *t* test and are presented as mean ± SD. *I*, representative immunofluorescence micrographs showing increased neutral lipids in the cartilage of metatarsal organ cultures *ex vivo* after a 10-days treatment with 5 ng/ml TGF-β2. BODIPY 493/503 visualizes neutral lipids (*green*), and Dapi labels nuclei (*blue*). Images are representative of six independent experiments (n = 6). Scale bars: 20 μm (first three columns) and 10 μm (last column, enlarged images). *J*, mean fluorescence intensity quantification validating the observed neutral lipid abundance in (i). Data from 12 replicates of six independent experiments (n = 6) were analyzed by unpaired two-tailed Student's *t* test and are presented as mean ± SD.

To dissect how TGF-β2 regulates chondrocyte metabolism, RNA sequencing was first performed in chondrocytes with/without treatment of TGF-β2 at 5 ng/ml for 24 h (We have completed dose screening over a range of 0–10 ng/ml for chondrocyte viability, migration and proliferation (35)). As shown in the volcano plot, TGF-β2 triggered a substantial change in the transcriptome of chondrocytes, leading to the upregulation of 286 transcripts and downregulation of 245 transcripts (Fig. 1*A*, fold change > 1.5 & *p* < 0.05). The bubble chart demonstrated that TGF-β2 extensively regulated lipid metabolism-related pathways, including glycosphingolipid biosynthesis (lacto and neolacto series), PPAR signaling, biosynthesis of unsaturated fatty acids, ether lipid metabolism, sphingolipid metabolism, fatty acid metabolism, steroidogenesis and phospholipase D signaling (Fig. 1*B*), which are integral to intracellular lipid droplet synthesis and overall lipid metabolism (36). Categorization of TGF-β2-induced metabolic genes further indicated that lipid metabolism-related candidates were the most prevalent, accounting for 42.02% of the total, surpassing other functional categories such as carbohydrate metabolism, amino acid metabolism and mitochondrial function (Fig. 1*C*).

To track intracellular lipid droplet formation in living chondrocytes, we first applied a label-free real-time cell monitoring system (Fig. 1*D* and Videos S1, S2). The results showed that TGF-β2 promoted chondrocytes to form a greater number of dynamic lipid droplets. Quantitative analyses confirmed the increase in lipid droplets in living chondrocytes induced by TGF-β2 at 5 ng/ml (Fig. 1*E*). To visualize and quantify lipid droplets in chondrocytes, we then applied BODIPY 493/503 to label neutral lipids and lipid droplets as described (16, 37). The results revealed that TGF-β2 significantly increased neutral lipids and lipid droplet accumulation in individual chondrocytes (Fig. 1*F*) and a collective of chondrocytes (Fig. S1) relative to the untreated controls. Quantitative analysis confirmed that TGF-β2 enhanced neutral lipid accumulation up to ∼2.0-fold (Fig. 1*G*) and increased lipid droplet number up to ∼4.3-fold (Fig. 1*H*) relative to untreated chondrocytes. To directly assess the content changes of neutral lipids in chondrocytes induced by TGF-β2, we detected intracellular triglyceride and cholesteryl ester contents, the major components of neutral lipids (38), and found that both of them were significantly increased in chondrocytes induced by TGF-β2 compared with the control groups (Fig. S2). Consistent with our observations at the cellular level *in vitro*, we next applied metatarsal organ cultures *ex vivo* to investigate the effect of TGF-β2 on tissue lipid synthesis (Fig. 1*I*). After induction with TGF-β2 at 5 ng/ml for 10 days, we found that TGF-β2 induced a significant surge in neutral lipids in cartilage layers (The yellow arrows indicated). Quantitative analysis based on fluorescence intensity confirmed the results (Fig. 1*J*).

Given the significant increase of TGF-β2 in osteoarthritic cartilage, especially in chondrocytes of deeper cartilage layers in tandem with escalating osteoarthritis severity (28, 29), we further detected the changes of neutral lipids in osteoarthritic cartilage (Fig. S3*A*). The results showed that neutral lipids were significantly accumulated in osteoarthritic cartilage layers, particularly in cartilaginous osteoid-like regions where the mineralization marker osterix (Osx) was highly expressed (39). Quantitative analysis based on fluorescence intensity confirmed the results (Fig. S3*B*). The enhanced lipid accumulation in osteoarthritic cartilage suggests the potential contribution of TGF-β2 to cartilage lipid metabolism disorder.

### TGF-β2 promotes lipid droplet accumulation *via* Fabp5

**Figure 2:**
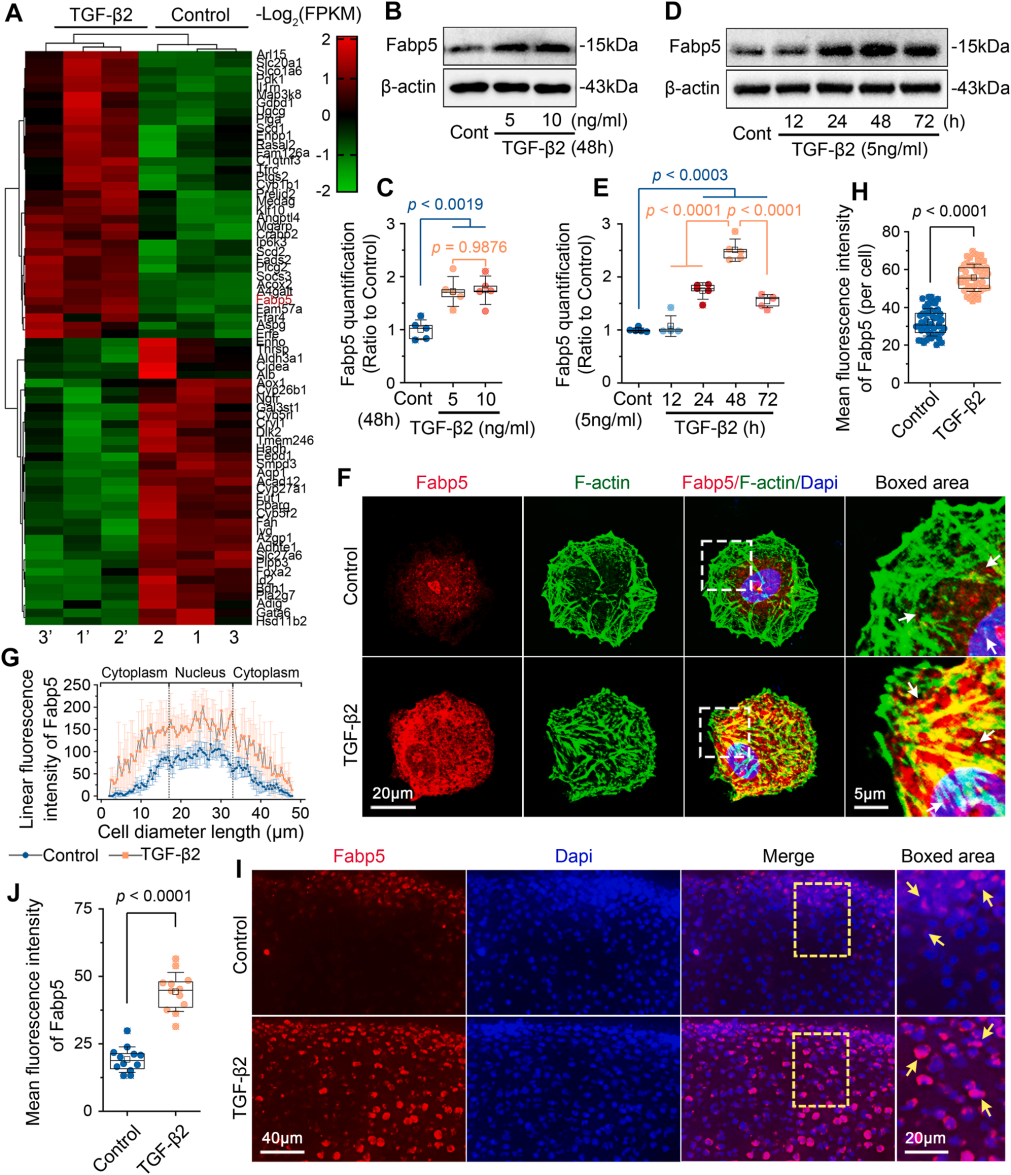
**TGF-β2 upregulates Fabp5 expression.***A*, pheatmap illustrating the cluster of differentially expressed genes involved in lipid metabolism in chondrocytes treated with 5 ng/ml TGF-β2 for 24 h. Samples 1 and 1′, 2 and 2′, 3 and 3′ are derived from the same mother cells. *B*, Western blotting showing that treatment with TGF-β2 at 5 and 10 ng/ml significantly increased Fabp5 expression in chondrocytes. Images are representative of five independent experiments (n = 5). *C*, quantitative analysis confirming the fold changes of Fabp5 in (*B*). Data from five independent experiments (n = 5) were analyzed by one-way ANOVA and are presented as mean ± SD. *D*, Western blotting showing that treatment with TGF-β2 at 5 ng/ml for 12, 24, 48, and 72 h significantly increased Fabp5 expression in chondrocytes. Images are representative of five independent experiments (n = 5). *E*, quantitative analysis confirming the fold changes of Fabp5 in (*D*). Data from five independent experiments (n = 5) were analyzed by one-way ANOVA and are presented as mean ± SD. *F*, representative immunofluorescence micrographs showing a significant increase in Fabp5 expression in the cytoplasm and nuclei of chondrocytes after a 48-h incubation with 5 ng/ml TGF-β2. Fabp5 is labeled in *red* (Alexa Fluor 647). F-actin cytoskeleton is visualized with FITC-phalloidin (*green*), and nuclei are stained with Dapi (*blue*). Images are representative of six independent experiments (n = 6). Scale bars: 20 μm (first three columns) and 5 μm (last column, enlarged images). *G*, linear fluorescence intensity quantification validating the distribution and quantity of Fabp5 in chondrocytes in (*F*). Data from 5 individual cell replicates of six independent experiments (n = 6) are presented as mean ± SD. *H*, mean fluorescence intensity quantification per chondrocyte confirming the Fabp5 abundance in (*F*). Data from 50 individual cell replicates of six independent experiments (n = 6) were analyzed by unpaired two-tailed Student's *t* test and are presented as mean ± SD. *I*, representative immunofluorescence micrographs showing increased Fabp5 expression in the cartilage of *ex vivo* metatarsal organ cultures after a 10-days treatment with 5 ng/ml TGF-β2. Fabp5 is labeled in *red* (Alexa Fluor 647), and nuclei are stained with Dapi (*blue*). Images are representative of six independent experiments (n = 6). Scale bars: 40 μm (first three columns) and 20 μm (last column, enlarged images). *J*, mean fluorescence intensity quantification validating the observed Fabp5 abundance in (*i*). Data from 12 replicates of six independent experiments (n = 6) were analyzed by unpaired two-tailed Student's *t* test and are presented as mean ± SD.

To explore the regulators of lipid droplet formation in chondrocytes in response to TGF-β2, we clustered all gene candidates potentially associated with intracellular lipid accumulation by a pheatmap (Fig. 2*A*). Notably, we revealed that TGF-β2 induced a significant upregulation of Fabp5 (its *p*-value ranks second lowest among all candidates), a lipid chaperone intricately linked to lipid droplet dynamics through facilitating lipid transport (40), enhancing lipid droplet biogenesis (41), and suppressing lipid oxidation (42). We next investigated the expression of Fabp5 in chondrocytes in response to TGF-β2 at different concentrations and time durations by western blotting (Fig. 2, *B–E*). We observed a significant increase in Fabp5 protein, up to ∼1.7-fold compared with the normal control group, in chondrocytes treated with TGF-β2 at 5 and 10 ng/ml for 48 h (Fig. 2, *B* and *C*). At a concentration of 5 ng/ml, we found that TGF-β2 increased the expression of Fabp5 (up to ∼2.5-fold) relative to the normal control group within the 72 h treatment period (Fig. 2, *D* and *E*). We next detected the intracellular distribution and expression of Fabp5 in chondrocytes by immunofluorescence staining. The results showed that Fabp5 was predominantly distributed in the cytoplasmic and nuclear regions, and TGF-β2 notably increased Fabp5 expression in both regions of individual chondrocytes (Fig. 2*F*) and a collective of chondrocytes (Fig. S4). The increase in Fabp5 expression was further confirmed by both linear and mean fluorescence intensity quantification per cell (Fig. 2, *G* and *H*). Consistent with our cellular observations *in vitro*, *ex vivo* metatarsal organ cultures treated with TGF-β2 at 5 ng/ml for 10 days showed increased expression of Fabp5 in cartilage layers (Fig. 2, *I* and *J*). Moreover, in osteoarthritic cartilage layers, we found that the expression of Fabp5 was enhanced, particularly in cartilaginous osteoid-like regions where Osx was highly expressed (Fig. S5).

**Figure 3:**
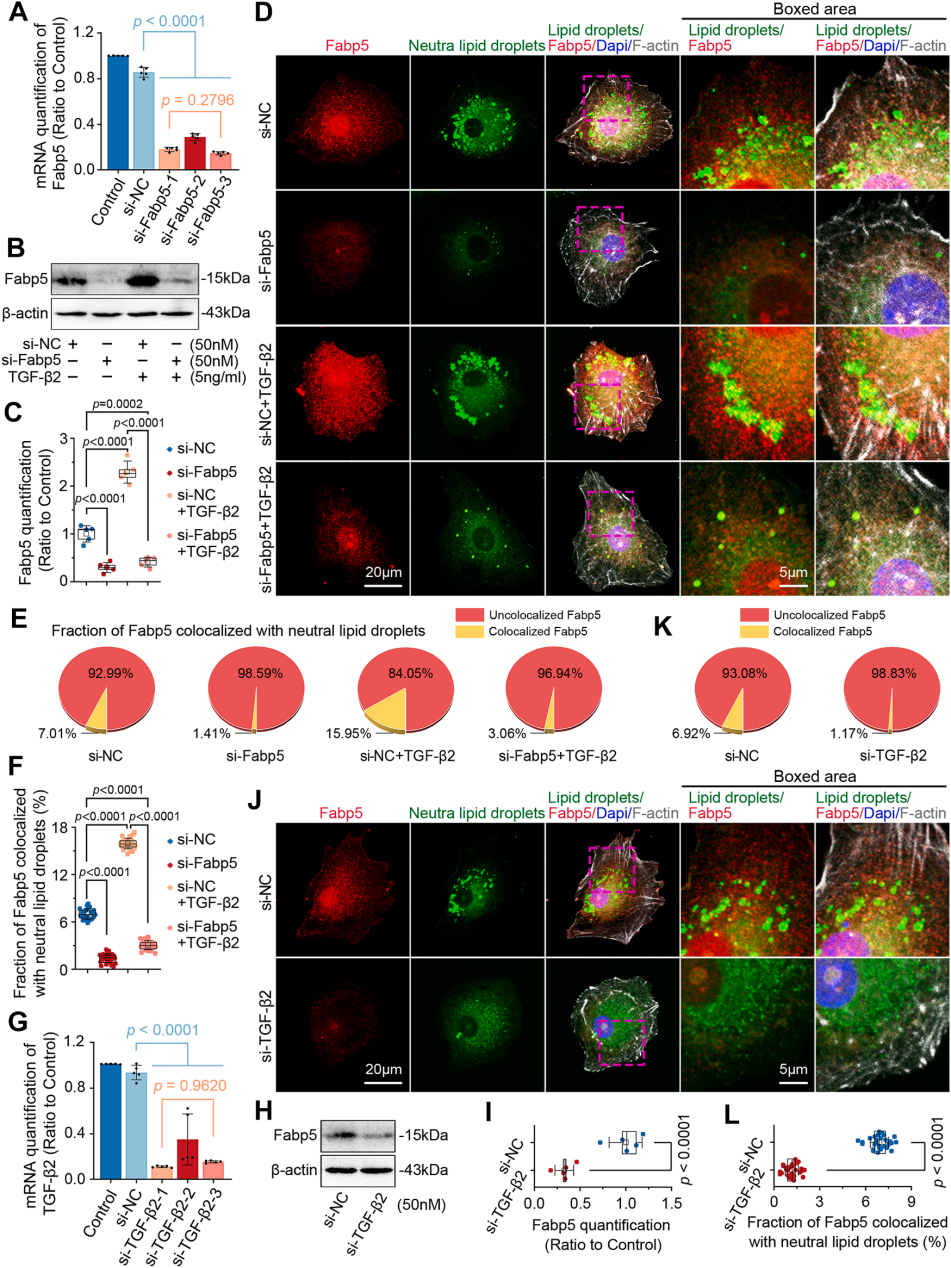
**Fabp5 is a vital regulator in TGF-β2-induced lipid droplet accumulation.***A*, qPCR showing that transfection with si-Fabp5-3 at 50 nM for 24 h efficiently knocked Fabp5 down in chondrocytes. Hprt served as the endogenous reference gene. Data from five independent experiments (n = 5) were analyzed by one-way ANOVA and are presented as mean ± SD. *B* and *C*, Western blotting (*B*) and quantification (*C*) showing that transfection with si-Fabp5-3 at 50 nM significantly decreased Fabp5 protein expression in chondrocytes. Images are representative of five independent experiments (n = 5). Corresponding quantitative data were analyzed by one-way ANOVA and are presented as mean ± SD. *D*, representative immunofluorescence micrographs showing that Fabp5 knockdown decreased neutral lipids and lipid droplets in chondrocytes in the presence of TGF-β2 at 5 ng/ml for 48 h. Neutral lipids and lipid droplets are labeled with BODIPY 493/503 (*green*), Fabp5 is visualized in *red* (Alexa Fluor 647), F-actin cytoskeleton is visualized with TRITC-phalloidin (*gray*, Em565), and nuclei are labeled with Dapi (*blue*). Images are representative of six independent experiments (n = 6). Scale bars: 20 μm (first three columns) and 5 μm (last two columns, enlarged images). *E* and *F* Pie chart (*E*) and box plot (*F*) showing the quantitative results of the observed Fabp5-lipids/lipid droplets colocalization in (*D*). Data from 30 individual cell replicates of six independent experiments (n = 6) were analyzed by one-way ANOVA and are presented as mean ± SD. *G*, qPCR showing that transfection with si-TGF-β2-1 at 50 nM for 24 h efficiently knocked TGF-β2 down in chondrocytes. Hprt served as the endogenous reference gene. Data from five independent experiments (n = 5) were analyzed by one-way ANOVA and are presented as mean ± SD. *H* and *I*, Western blotting (*H*) and quantification (*I*) showing that transfection with si-TGF-β2-1 at 50 nM significantly decreased Fabp5 protein expression in chondrocytes. Images are representative of five independent experiments (n = 5). Corresponding quantitative data were analyzed by unpaired two-tailed Student's *t* test and are presented as mean ± SD. *J*, Representative immunofluorescence micrographs showing that TGF-β2 knockdown decreased neutral lipids and lipid droplets in chondrocytes. Neutral lipids and lipid droplets are labeled with BODIPY 493/503 (*green*), Fabp5 is visualized in *red* (Alexa Fluor 647), F-actin cytoskeleton is visualized with TRITC-phalloidin (*gray*, Em565), and nuclei are labeled with Dapi (*blue*). Images are representative of six independent experiments (n = 6). Scale bars: 20 μm (first three columns) and 5 μm (last two columns, enlarged images). *K* and *L* Pie chart (*K*) and box plot (*L*) showing the quantitative results of the observed Fabp5-lipids/lipid droplets colocalization in (*J*). Data from 30 individual cell replicates of six independent experiments (n = 6) were analyzed by unpaired two-tailed Student's *t* test and are presented as mean ± SD.

To validate the importance of Fabp5 in lipid droplet accumulation induced by exogenous TGF-β2, we conducted siRNA transfection in chondrocytes. After validating the knockdown efficacy of siRNAs targeting Fabp5 by the quantitative real-time polymerase chain reaction (qPCR) (Fig. 3*A*), we chose to use si-Fabp5-3, which exhibited the highest knockdown efficiency (∼85.3%), in subsequent loss-of-function experiments. By western blotting, we found that Fabp5 knockdown significantly suppressed the expression of Fabp5 protein in chondrocytes even in the presence of TGF-β2 (Fig. 3, *B* and *C*). By immunofluorescence staining, we detected that Fabp5 knockdown effectively decreased neutral lipids and lipid droplet accumulation in chondrocytes in the presence of TGF-β2 (Fig. 3*D*). Quantitative analysis of Fabp5 expression (Fig. S6*A*), intracellular lipids (Fig. S6*B*), and lipid droplet number (Fig. S6*C*) confirmed the results. By using triglyceride and cholesteryl ester kits, we directly detected the intracellular content changes of triglycerides and cholesteryl esters in chondrocytes induced by si-Fabp5 in the presence of TGF-β2 at 5 ng/ml (Fig. S7). These data showed that Fabp5 knockdown directly decreased neutral lipid contents (triglycerides and cholesteryl esters) in chondrocytes in the presence of TGF-β2. As Fabp5 can accumulate on the surface of lipid droplets to influence lipid droplet formation and deposition (40, 41, 43), we performed an intracellular colocalization analysis between Fabp5 and lipid droplets. The results demonstrated that Fabp5 knockdown severely impaired its colocalization with lipid droplets, reducing the rate from ∼7.0% (non-targeting control small interfering RNA (si-NC)) to ∼1.4% (si-Fabp5) and, in the presence of TGF-β2, from ∼16.0% (si-NC + TGF-β2) to ∼3.1% (si-Fabp5+TGF-β2). These results validated the critical role of Fabp5 in mediating TGF-β2-promoted lipid droplet accumulation (Fig. 3, *E* and *F*).

To determine whether endogenous TGF-β2 also participates in the lipid droplet accumulation through Fabp5, we knocked down TGF-β2 in chondrocytes. The results from siRNAs targeting TGF-β2 showed that endogenous TGF-β2 was efficiently knocked down, especially in si-TGF-β2-1 groups (Fig. 3*G*, knockdown efficiency up to ∼88.9%). Western blotting revealed that endogenous TGF-β2 knockdown significantly decreased the protein expression of Fabp5 in chondrocytes (Fig. 3, *H* and *I*). Immunofluorescence staining demonstrated that TGF-β2 knockdown suppressed both Fabp5 expression and lipid droplet accumulation (Fig. 3*J*). Quantitative analysis of Fabp5 expression (Fig. S8*A*), intracellular lipids (Fig. S8*B*), and lipid droplet number (Fig. S8*C*) confirmed these results. Endogenous TGF-β2 knockdown substantially reduced the colocalization of Fabp5 with lipid droplets in chondrocytes from 6.9% to 1.2%, demonstrating the importance of basal TGF-β2 signaling in Fabp5-mediated lipid droplet accumulation (Fig. 3, *K* and *L*). We next detected the changes in intracellular triglyceride and cholesteryl ester contents in chondrocytes induced by si-TGF-β2 and found that endogenous TGF-β2 knockdown decreased the contents of triglycerides and cholesteryl esters in chondrocytes (Fig. S9).

Collectively, these data indicate that Fabp5 plays a vital role in TGF-β2-mediated neutral lipid accumulation and lipid droplet formation in chondrocytes.

### TGF-β2-enhanced lipid droplet accumulation requires the participation of TβRI

**Figure 4:**
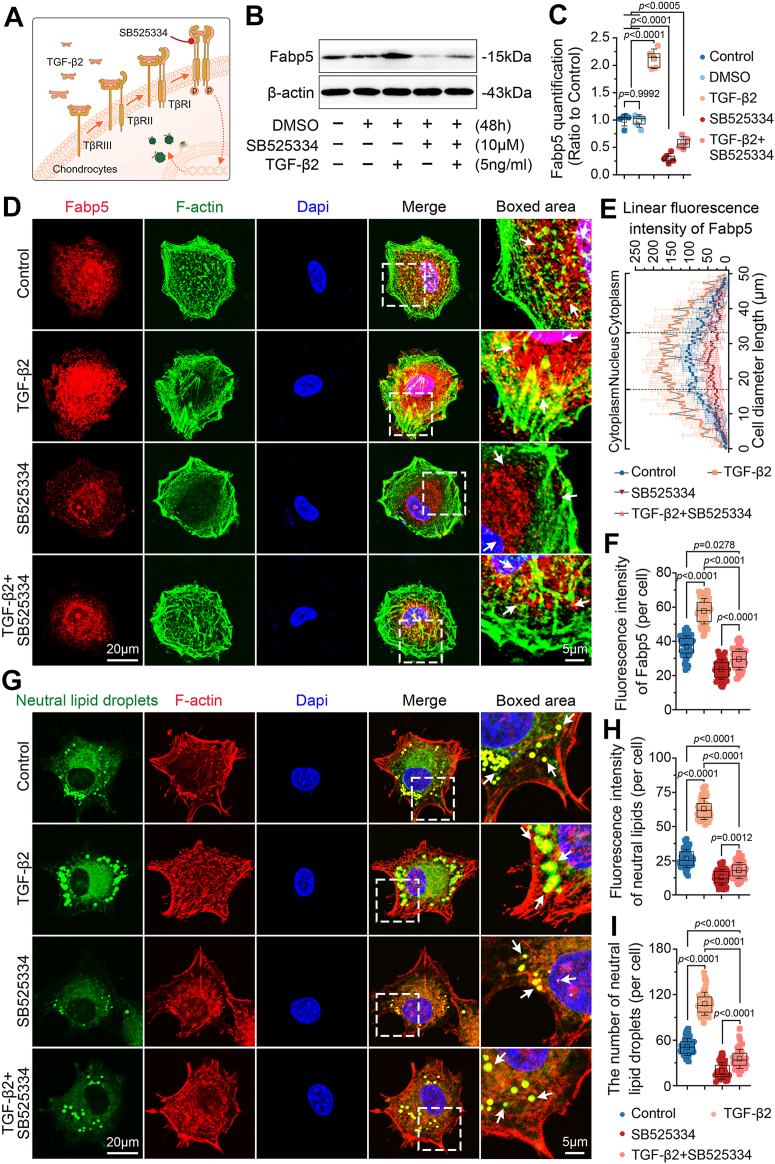
**TGF-β2 promotes lipid droplet accumulation *via* TβRI.***A*, schematic depicting TβRI-dependent signaling cascades initiated by TGF-β2 [57]. *B* Western blotting showing that TβRI inhibition with SB525334 decreased the expression of Fabp5 in chondrocytes in the presence of TGF-β2 (5 ng/ml). Data are representative of five independent experiments (n = 5). *C*, quantitative analysis confirming the fold changes of Fabp5 in (*B*). Data from five independent experiments (n = 5) were analyzed by one-way ANOVA and are presented as mean ± SD. *D*, Representative immunofluorescence micrographs showing the reduction of Fabp5 protein in chondrocytes induced by SB525334 in the presence of TGF-β2 (5 ng/ml). Fabp5 is visualized in *red* (Alexa Fluor 647), F-actin cytoskeleton is labeled with FITC-phalloidin (*green*), and nuclei are labeled with Dapi (*blue*). Images are representative of five independent experiments (n = 5). Scale bars: 20 μm (first four columns) and 5 μm (last column, enlarged images). *E*, linear fluorescence intensity quantification validating the distribution and quantity of Fabp5 in chondrocytes in (*D*). Data from 5 individual cell replicates of five independent experiments (n = 5) are presented as mean ± SD. *F*, mean fluorescence intensity quantification per chondrocyte confirming Fabp5 abundance in (*D*). Data from 50 individual cell replicates of five independent experiments (n = 5) were analyzed by one-way ANOVA and are presented as mean ± SD. *G*, representative immunofluorescence micrographs showing a significant reduction in lipids and lipid droplets in chondrocytes after treatment with SB525334 in the presence of TGF-β2 (5 ng/ml). BODIPY 493/503 visualizes neutral lipids and lipid droplets (*green*), TRITC-phalloidin delineates F-actin cytoskeleton (*red*), and Dapi labels nuclei (*blue*). Images are representative of five independent experiments (n = 5). Scale bars: 20 μm (first four columns) and 5 μm (last column, enlarged images). *H*, mean fluorescence intensity quantification per chondrocyte validating the observed neutral lipid abundance in (*G*). Data from 50 individual cell replicates of five independent experiments (n = 5) were analyzed by one-way ANOVA and are presented as mean ± SD. *I*, quantitative analysis of the lipid droplet number per chondrocyte confirming the observed lipid droplet abundance in (*G*). Data from 50 individual cell replicates of five independent experiments (n = 5) were analyzed by one-way ANOVA and are presented as mean ± SD.

As illustrated in Figure 4*A*, canonical initiation of TGF-β signaling involves ligand binding to the constitutively active receptor TβRII, often facilitated by the accessory receptor TβRIII. This event triggers the recruitment and phosphorylation of the indispensable signal-transducing receptor TβRI, forming an active heteromeric complex. The activated TβRI subsequently serves as the key kinase to initiate the downstream signaling cascade (44). To determine the role of TβRI in TGF-β2-induced lipid droplet accumulation in chondrocytes, we firstly applied SB525334, a specific inhibitor for TβRI (45). We found that SB525334 could effectively decrease the expression of Fabp5 protein in chondrocytes in the presence of TGF-β2 at 5 ng/ml (Fig. 4*B*). Quantitative analysis confirmed the effect of SB525334 on Fabp5 (Fig. 4*C*). By immunofluorescence staining, we detected the distribution change of Fabp5 in individual chondrocytes (Fig. 4*D*) and a collective of chondrocytes (Fig. S10). The results showed that SB525334 could decrease the expression of Fabp5 protein in chondrocytes in the presence of TGF-β2. Quantitative analysis of linear and mean fluorescence intensity confirmed this result (Fig. 4, *E* and *F*). The above results indicate that SB525334 not only acts as a specific inhibitor of the TβRI, but also has a direct regulatory effect on Fabp5 expression. We next investigated the importance of TβRI in TGF-β2-increased neutral lipids and lipid droplet accumulation in chondrocytes. From immunofluorescence staining, we observed that TβRI inhibition by SB525334 could effectively reduce neutral lipids and lipid droplet accumulation in individual chondrocytes (Fig. 4*G*) and a collective of chondrocytes (Fig. S11) in the presence of exogenous TGF-β2. Quantitative analysis of the neutral lipids per cell (Fig. 4*H*) and lipid droplet number (Fig. 4*I*) further confirmed the results. By using triglyceride and cholesteryl ester kits, we then detected the specific intracellular changes of triglycerides and cholesteryl esters in chondrocytes induced by SB525334 in the presence of TGF-β2 (Fig. S12). The results showed that TβRI inhibition by SB525334 directly decreased the main lipid contents, triglycerides and cholesteryl esters, in chondrocytes induced by exogenous TGF-β2. Collectively, these results indicate that TβRI plays a key role in TGF-β2-increased neutral lipids and lipid droplet accumulation in chondrocytes.

### TGF-β2 promotes lipid droplet accumulation *via* TβRI/p-Smad3 signaling

**Figure 5:**
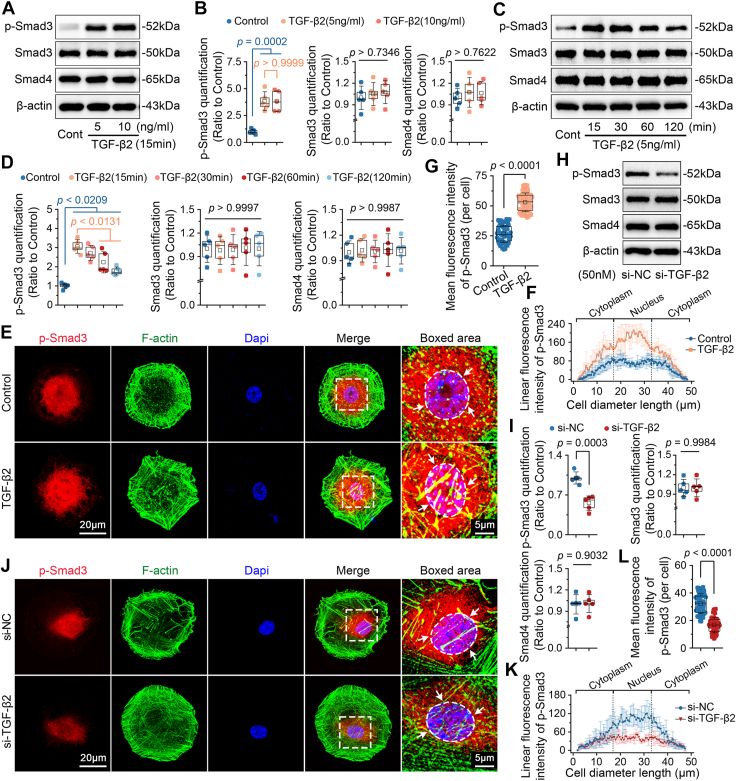
**TGF-β2 activates p-Smad3 signaling.***A*, Western blotting showing that treatment with TGF-β2 at 5 and 10 ng/ml for 15 min significantly increased p-Smad3 expression in chondrocytes. Images are representative of five independent experiments (n = 5). *B*, Quantitative analysis confirming the fold changes of p-Smad3 in (*A*). Data from five independent experiments (n = 5) were analyzed by one-way ANOVA and are presented as mean ± SD. *C*, Western blotting showing that treatment with TGF-β2 at 5 ng/ml for 15, 30, 60, and 120 min significantly increased p-Smad3 expression in chondrocytes. Images are representative of five independent experiments (n = 5). *D*, quantitative analysis confirming the fold changes of p-Smad3 in (*C*). Data from five independent experiments (n = 5) were analyzed by one-way ANOVA and are presented as mean ± SD. *E*, representative immunofluorescence micrographs showing a significant increase of p-Smad3 expression in the cytoplasmic and nuclei of chondrocytes incubated with 5 ng/ml TGF-β2 for 15 min p-Smad3 is labeled in red (Alexa Fluor 647), F-actin cytoskeleton is visualized with FITC-phalloidin (*green*), and nuclei are stained with Dapi (*blue*). Images are representative of six independent experiments (n = 6). Scale bars: 20 μm (first four columns) and 5 μm (last column, enlarged images). *F*, linear fluorescence intensity quantification per chondrocyte validating the distribution and quantity of p-Smad3 in chondrocytes in (*E*). Data from 5 individual cell replicates of six independent experiments (n = 6) are presented as mean ± SD. *G*, mean fluorescence intensity quantification per chondrocyte confirming p-Smad3 abundance in (*E*). Data from 50 individual cell replicates of six independent experiments (n = 6) were analyzed by unpaired two-tailed Student's *t* test and are presented as mean ± SD. *H*, Western blotting showing that knockdown endogenous TGF-β2 significantly decreased p-Smad3 expression in chondrocytes. Images are representative of five independent experiments (n = 5). *I*, quantitative analysis confirming the fold changes of p-Smad3 in (*H*). Data from five independent experiments (n = 5) were analyzed by unpaired two-tailed Student's *t* test and are presented as mean ± SD. *J*, Representative immunofluorescence micrographs showing that knockdown endogenous TGF-β2 induced a substantial reduction in p-Smad3 protein in chondrocytes. p-Smad3 is labeled in red (Alexa Fluor 647), F-actin cytoskeleton is visualized with FITC-phalloidin (*green*), and nuclei are stained with Dapi (*blue*). Images are representative of five independent experiments (n = 5). Scale bars: 20 μm (first four columns) and 5 μm (last column, enlarged images). *K*, linear fluorescence intensity quantification per chondrocyte validating the distribution and quantity of p-Smad3 in chondrocytes in (*J*). Data from 5 individual cell replicates of five independent experiments (n = 5) are presented as mean ± SD. *L*, Mean fluorescence intensity quantification per chondrocyte confirming p-Smad3 abundance in (*J*). Data from 50 individual cell replicates of five independent experiments (n = 5) were analyzed by unpaired two-tailed Student's *t* test and are presented as mean ± SD.

To investigate the immediate signaling response to TGF-β2 in chondrocytes, we next focused on the TGF-β signaling effector Smad3/4 complex, which mediates the canonical TGF-β pathway (35). Western blotting demonstrated that a 15-min exposure to 5 and 10 ng/ml TGF-β2 immediately upregulated phosphorylated-Smad3 (p-Smad3) in chondrocytes, while total Smad3 and Smad4 remained unchanged (Fig. 5, *A* and *B*). Time-course analysis revealed that TGF-β2 elicited a rapid p-Smad3 response, peaking at an early time point (15 min) with a ∼3.1-fold increase compared to the normal control group (Fig. 5, *C* and *D*). Immunofluorescence staining revealed that TGF-β2 enhanced the expression of p-Smad3 and triggered its nuclear accumulation in individual chondrocytes (Fig. 5*E*) and a collective of chondrocytes (Fig. S13). This effect was confirmed by quantitative analysis of linear and mean fluorescent intensity (Fig. 5, *F* and *G*). For the effect of endogenous TGF-β2 on p-Smad3 signaling, we revealed that its knockdown by siRNA also decreased the expression of p-Smad3 but did not induce the total protein changes of Smad3 and 4 (Fig. 5, *H* and *I*). Moreover, knockdown of endogenous TGF-β2 also impaired the expression of both cytoplasmic and nuclear p-Smad3 (Fig. 5*J*). Quantification of linear and mean fluorescence intensity further confirmed the observation (Fig. 5, *K* and *L*).

**Figure 6:**
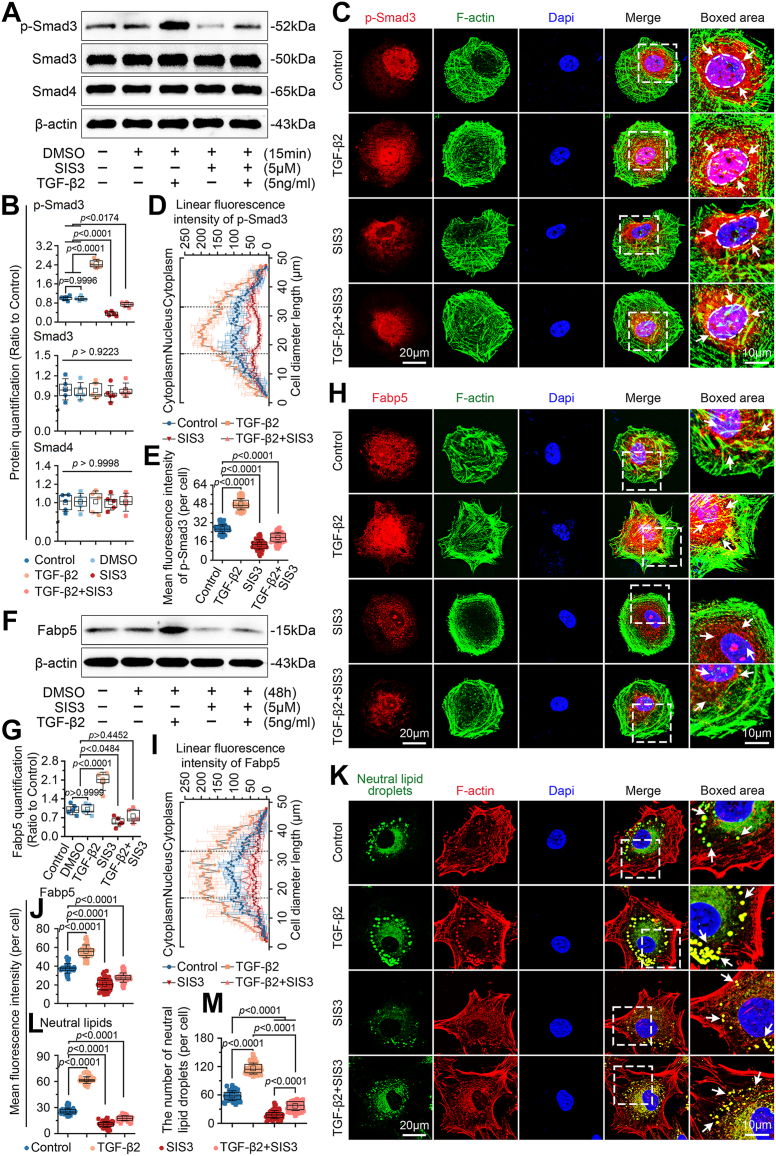
**TGF-β2-induced lipids/lipid droplets in chondrocytes depend on p-Smad3 signaling.***A*, Western blotting showing that the inhibitor SIS3 largely decreased p-Smad3 expression in chondrocytes in the presence of TGF-β2 (5 ng/ml). Data are representative of five independent experiments (n = 5). *B*, quantitative analysis confirming the fold changes of p-Smad3 in (*A*). Data from five independent experiments (n = 5) were analyzed by one-way ANOVA and are presented as mean ± SD. *C*, representative immunofluorescence micrographs showing that SIS3 induced a substantial reduction of p-Smad3 in chondrocytes in the presence of TGF-β2 (5 ng/ml). p-Smad3 is labeled in *red* (Alexa Fluor 647), F-actin cytoskeleton is labeled with FITC-phalloidin (*green*), and nuclei are stained with Dapi (*blue*). Images are representative of five independent experimental replicates (n = 5). Scale bars: 20 μm (first four columns) and 10 μm (last column, enlarged images). *D*, linear fluorescence intensity quantification per chondrocyte validating the distribution and quantity of p-Smad3 in chondrocytes in (*C*). Data from 5 individual cell replicates of five independent experiments (n = 5) are presented as mean ± SD. *E*, mean fluorescence intensity quantification per chondrocyte confirming p-Smad3 abundance in (*C*). Data from 50 individual cell replicates of five independent experiments (n = 5) were analyzed by one-way ANOVA and are presented as mean ± SD. *F*, Western blotting showing that p-Smad3 inhibition reduced Fabp5 expression in chondrocytes in the presence of TGF-β2 (5 ng/ml). Data are representative of five independent experiments (n = 5). *G*, quantitative analysis confirming the fold changes of Fabp5 in (*F*). Data from five independent experiments (n = 5) were analyzed by one-way ANOVA and are presented as mean ± SD. *H*, representative immunofluorescence micrographs showing that p-Smad3 inhibition decreased the expression of Fabp5 in chondrocytes in the presence of TGF-β2 (5 ng/ml). Fabp5 is labeled in red (Alexa Fluor 647), F-actin cytoskeleton is labeled with FITC-phalloidin (*green*), and nuclei are stained with Dapi (*blue*). Images are representative of five independent experiments (n = 5). Scale bars: 20 μm (first four columns) and 10 μm (last column, enlarged images). *I*, linear fluorescence intensity quantification per chondrocyte validating the distribution and quantity of Fabp5 in chondrocytes in (*H*). Data from 5 individual cell replicates of five independent experiments (n = 5) are presented as mean ± SD. *J*, mean fluorescence intensity quantification per chondrocyte confirming Fabp5 abundance in (*H*). Data from 50 individual cell replicates of five independent experiments (n = 5) were analyzed by one-way ANOVA and are presented as mean ± SD. *K*, representative immunofluorescence micrographs showing that p-Smad3 inhibition decreased neutral lipids and lipid droplets in chondrocytes in the presence of TGF-β2 (5 ng/ml). BODIPY 493/503 visualizes neutral lipids and lipid droplets (*green*), TRITC-phalloidin delineates F-actin cytoskeleton (*red*), and Dapi labels nuclei (*blue*). Images are representative of five independent experiments (n = 5). Scale bars: 20 μm (first four columns) and 10 μm (last column, enlarged images). *L*, mean fluorescence intensity quantification per chondrocyte validating the observed neutral lipid abundance in (*K*). Data from 50 individual cell replicates of five independent experiments (n = 5) were analyzed by one-way ANOVA and are presented as mean ± SD. *M*, quantitative analysis of the lipid droplet number per chondrocyte confirming the lipid droplet abundance in (*K*). Data from 50 individual cell replicates of five independent experiments (n = 5) were analyzed by one-way ANOVA and are presented as mean ± SD.

To determine the importance of p-Smad3 signaling in TGF-β2-induced lipid droplet accumulation, we applied SIS3, a specific inhibitor of p-Smad3 (46). By western blotting, we verified the inhibitory effects of SIS3 on p-Smad3, while total Smad3 and 4 remained stable (Fig. 6, *A* and *B*). By immunofluorescence staining, we demonstrated that SIS3 largely reduced p-Smad3 in individual chondrocytes (Fig. 6*C*) and a collective of chondrocytes (Fig. S14) in the presence of TGF-β2. This result was verified by quantitative analysis of linear and mean fluorescence intensity per cell (Fig. 6, *D* and *E*). Subsequently, we explored the effects of p-Smad3 inhibition on the expression of Fabp5 protein. Western blotting revealed that SIS3 reduced the expression of Fabp5 protein in chondrocytes in the presence of TGF-β2 (Fig. 6, *F* and *G*). Immunofluorescence staining demonstrated that SIS3 induced a decrease in Fabp5 in both cytoplasmic and nuclear regions of individual chondrocytes (Fig. 6*H*) and a collective of chondrocytes (Fig. S15) in the presence of TGF-β2, and quantitative analysis of linear and mean fluorescence intensity validated the observation (Fig. 6, *I* and *J*). Ultimately, we investigated the effects of p-Smad3 inhibition on lipid droplet accumulation. From immunofluorescence staining, we found that SIS3 greatly decreased the intracellular accumulation of neutral lipids and lipid droplets in individual chondrocytes (Fig. 6*K*) and a collective of chondrocytes (Fig. S16) in the presence of TGF-β2. Quantitative analysis of neutral lipids and lipid droplets further validated the observation (Fig. 6, *L* and *M*). To further show the content changes in neutral lipids, we detected triglycerides and cholesteryl esters in chondrocytes induced by SIS3 in the presence of TGF-β2 (Fig. S17). The results showed that inhibition of p-Smad3 signaling decreased the contents of triglycerides and cholesteryl esters in chondrocytes in the presence of TGF-β2, indicating the importance of p-Smad3 signaling in TGF-β2-regulated neutral lipids and lipid droplets in chondrocytes.

**Figure 7:**
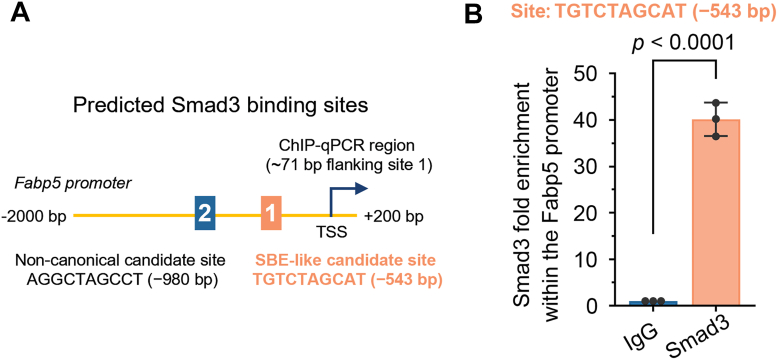
**Activated Smad3 directly binds to the promoter region of Fabp5 in chondrocytes.***A*, schematic representation of the mouse Fabp5 promoter region (−2000 bp to +200 bp relative to TSS) showing the two predicted Smad3 motif matches identified by JASPAR analysis: Site 1, TGTCTAGCAT (chr3:10077102–10077111, mm39; approximately −543 to −534 bp), an SBE-like candidate site containing the GTCT core; and Site 2, AGGCTAGCCT (chr3:10076665–10076674, mm39; approximately −980 to −971 bp), a non-canonical/GC-rich candidate region. The position of the ChIP-qPCR amplification is indicated as “1” & “2”. *B*, ChIP-qPCR analysis showing the Smad3 enrichment at the SBE-like site of the promoter region of Fabp5 in chondrocytes compared with the IgG control. Data from three independent experiments (n = 3) were analyzed by unpaired two-tailed Student's *t* test and are presented as mean ± SD.

To determine whether nuclear p-Smad3 directly binds to the promoter region of Fabp5, we analyzed the mouse Fabp5 promoter (−2000 bp to +200 bp relative to the transcription start site (TSS)) using the JASPAR Smad3 matrix and identified two candidate Smad3 motif matches: a Smad-binding element (SBE)-like candidate site containing the GTCT core at approximately −543 to −534 bp, and a non-canonical/GC-rich candidate region at approximately −980 to −971 bp (Fig. 7*A*). From chromatin immunoprecipitation (ChIP)-qPCR results (Fig. 7*B*), we observed significant enrichment of p-Smad3 bound to the SBE-like site of the promoter region of Fabp5 in chondrocytes compared with the IgG control (∼40.15-fold enrichment, *p* < 0.0001), whereas no appreciable amplification was detected at the non-canonical GC-rich candidate region. Together with our evidence showing that increased nuclear p-Smad3 led to subsequent elevated Fabp5 expression (Figs. 5 and 6), these results collectively support that accumulated nuclear p-Smad3 directly binds to the SBE-like site of Fabp5 promoter region and contributes to Fabp5 transcriptional regulation.

**Figure 8:**
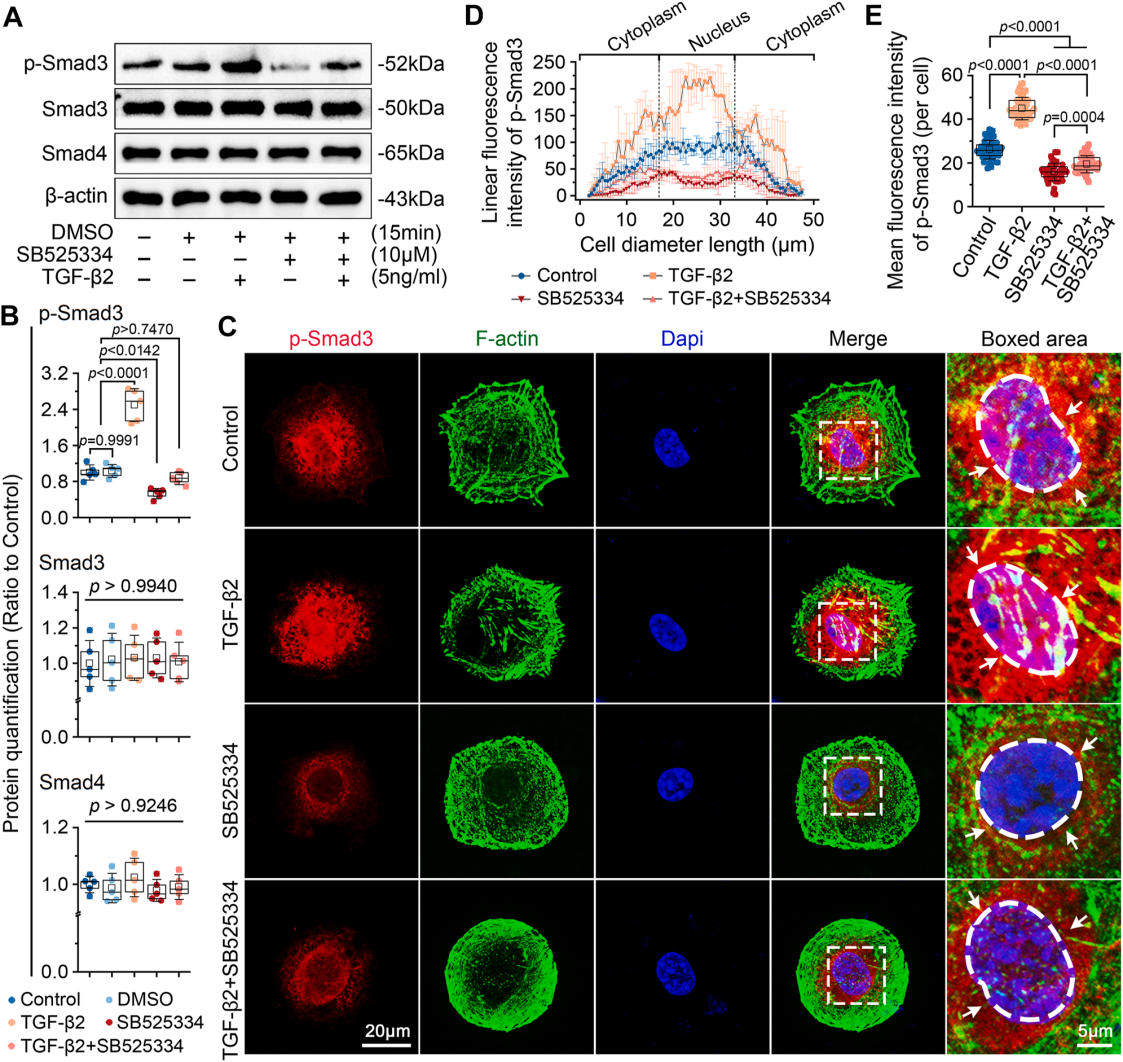
**TGF-β2 activates p-Smad3 signaling through TβRI.***A*, Western blotting showing that TβRI inhibition (SB525334) decreased the expression of p-Smad3 in chondrocytes in the presence of TGF-β2 at 5 ng/ml for 15 min. Data are representative of five independent experiments (n = 5). *B*, quantitative analysis confirming the fold changes of p-Smad3 in (*A*). Data from five independent experiments (n = 5) were analyzed by one-way ANOVA and are presented as mean ± SD. *C*, representative immunofluorescence micrographs showing that TβRI inhibition induced a substantial reduction in p-Smad3 in chondrocytes in the presence of 5 ng/ml TGF-β2 for 15 min p-Smad3 is labeled in *red* (Alexa Fluor 647), F-actin cytoskeleton is highlighted with FITC-phalloidin (*green*), and nuclei are stained with Dapi (*blue*). Images are representative of five independent experiments (n = 5). Scale bars: 20 μm (first four columns) and 5 μm (last column, enlarged images). *D*, Linear fluorescence intensity quantification per chondrocyte validating the distribution and quantity of p-Smad3 in chondrocytes in (*C*). Data from 5 individual cell replicates of five independent experiments (n = 5) are presented as mean ± SD. *E*, Mean fluorescence intensity quantification per chondrocyte confirming p-Smad3 abundance in (*C*). Data from 50 individual cell replicates of five independent experiments (n = 5) were analyzed by one-way ANOVA and are presented as mean ± SD.

To further establish a functional link between TβRI and p-Smad3 signaling, we detected the expression change of p-Smad3 in chondrocytes treated with the TβRI inhibitor SB525334 in the presence of TGF-β2. From western blotting, we found that SB525334 reduced the expression of p-Smad3 but did not change the expression of total Smad3 and 4 in chondrocytes in the presence of TGF-β2 (Fig. 8, *A* and *B*). From immunofluorescence staining, we found that SB525334 decreased the expression of p-Smad3 in both cytoplasmic and nuclear regions of individual chondrocytes (Fig. 8*C*) and a collective of chondrocytes (Fig. S18) in the presence of TGF-β2. This result was further validated by quantitative analysis of linear and mean fluorescence intensity (Fig. 8, *D* and *E*).

Collectively, these data indicate the importance of the TβRI/p-Smad3 axis in TGF-β2-mediated neutral lipid and lipid droplet accumulation in chondrocytes.

### Cell metabolomics reveals TGF-β2-driven enrichment of lipid metabolites involved in lipid droplet accumulation

**Figure 9:**
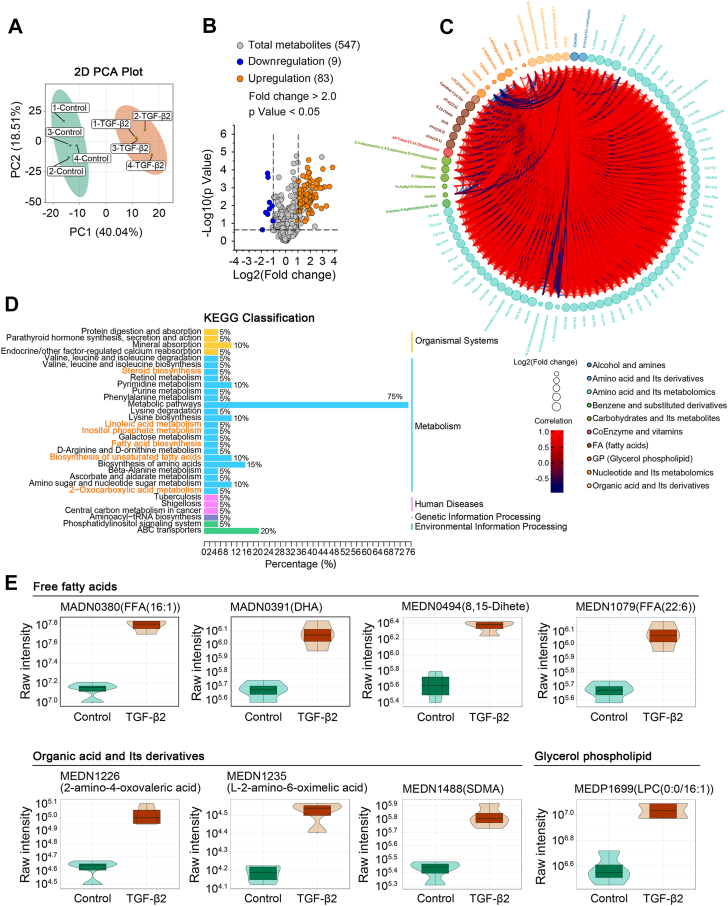
**Metabolomics reveals TGF-β2-driven enrichment of lipid metabolites involved in lipid droplet accumulation.***A*, 2D PCA plot revealing a clear separation between TGF-β2-treated (*orange circle*) and untreated (*green circle*) groups along the first principal component (PC1: 40.04%, PC2: 18.51%) (n = 4). *B*, Volcano plot highlighting significantly altered metabolites in chondrocytes treated with 5 ng/ml TGF-β2 for 48 h, with 83 metabolites upregulated and 9 metabolites downregulated. Fold change ≥ 2.0 & *p* < 0.05. *C*, chord diagram visualizing the relationships among significantly altered metabolite subtypes in chondrocytes in response to TGF-β2. *D*, Bar chart showing the KEGG pathway classification of altered metabolites. *E*, Violin plots showing the distribution and probability density of the most responsive lipid metabolites (*e*.*g*., free fatty acids, glycerol phospholipid, organic acid and its derivatives) in chondrocytes treated with 5 ng/ml TGF-β2 for 48 h.

To identify the metabolites involved in TGF-β2-mediated neutral lipid accumulation and lipid droplet formation, we conducted a wide-targeted metabolomic analysis. The 2D principal component analysis (PCA) plot revealed a clear separation between TGF-β2-treated and untreated groups along the first principal component (PC1), which accounted for 40.04% of the total variance (PC2: 18.51%), indicating a distinct global metabolic re-organization (Fig. 9*A*). The volcano plot analysis identified 91 significantly altered metabolites, with 82 metabolites upregulated and 9 metabolites downregulated (fold change ≥ 2.0 & *p* < 0.05), revealing a strong promotive effect of TGF-β2 on chondrocyte metabolites (Fig. 9*B*). Interrogation of the relationships among these differential metabolites using a chord diagram demonstrated coordinated changes in metabolite subtypes, mainly including but not limited to lipid metabolites (Fig. 9*C*). KEGG classification analysis further showed that these altered metabolites could be mainly enriched in 21 pathways (blue pillars), which belonged to four types of metabolism, *i*.*e*., amino acid metabolism (amino acid and its metabolites), lipid metabolism (fatty acyl compounds (FAs), organic acid and its derivatives), nucleotide metabolism (nucleotide and its metabolites), and carbohydrate metabolism (carbohydrates and its metabolites) (Figs. 9*D* and S19*A*). These altered 91 metabolites, along with one coenzyme and vitamin metabolite (all-trans-13,14-dihydroretinol) and one alcohol and amine (calcitriol), could be integrated into an interactive network through metabolite interaction (Fig. S19*B*). We analyzed the proportion of these metabolite categories and found that amino acid metabolism (amino acid and its metabolites) accounted for 71.7% (65 metabolites), lipid metabolism (FAs, organic acid and its derivatives) accounted for 15.3% (14 metabolites), nucleotide metabolism (nucleotide and its metabolites) accounts for 7.6% (7 metabolites), and carbohydrate metabolism (carbohydrates and its metabolites) accounts for 5.4% (5 metabolites) (Fig. S19*C*), which indicated that TGF-β2 largely affected amino acid metabolism, followed by lipid metabolism. In order to display the specific details of altered metabolites, we conducted cluster analysis on metabolites based on these four categories by pheatmaps (Fig. S19*D*). The results obviously showed the increase in metabolites in amino acid metabolism and lipid metabolism. We finally visualized representative metabolites selected from the top 50 altered metabolites using violin plots. There were 37 amino acid metabolites (the top 5 are shown in Fig. S19*E*, upper), 8 lipid metabolites (shown in Fig. 9*E*) and 4 nucleic acid metabolites and 1 carbohydrate metabolite (Fig. S19*E*, lower). Meanwhile, the violin plots also showed the changed metabolites in alcohol and amines (calcitriol) and coenzyme and vitamin (all-trans-13,14-dihydroretinol) in chondrocytes induced by TGF-β2 (Fig. S19*F*). In particular for lipid metabolism, it should be noted that intermediate metabolites involved in neutral lipid accumulation and lipid droplet formation were enriched in 6 pathways (Fig. 9*D*), and more importantly, fatty acid biosynthesis pathway and unsaturated fatty acid biosynthesis pathway, which are established components of lipid droplet formation (47, 48), were prominently enriched. The presented metabolic products, including free fatty acids, DHA, and glycerol phospholipids, are all involved in neutral lipid accumulation and lipid droplet formation, except for organic acid and its derivatives (49) (Fig. 9*E*). This evidence from lipid metabolites confirmed the regulation of TGF-β2 on chondrocyte lipids and lipid droplets.

## Discussion

Articular cartilage, though comprising less than 1% lipids by wet weight, contains a diverse lipid profile—including palmitic, oleic, and linoleic acids—critical for its structural and functional integrity (6, 7). In the avascular, aneural, and alymphatic microenvironment, chondrocytes rely on diffusion from synovial fluid and exchanges with subchondral bone to acquire lipids and other nutrients (29, 50, 51). These cells preserve lipid homeostasis by storing lipids in cytoplasmic lipid droplets and regulating their turnover, thereby supporting energy metabolism, signal transduction, protein synthesis, and DNA repair (7). Lipid droplet dynamics in chondrocytes are modulated by a complex interplay of biochemical and biomechanical cues within the osteochondral niche. Our prior work revealed that subchondral osteoblast-derived signals dysregulated the lipid metabolism enzyme profile, impaired cholesterol synthesis, and modified energy supply patterns in chondrocytes, yet it did not provide direct evidence regarding chondrocyte lipid droplet dynamics (52, 53, 54). Recent studies directly link aberrant lipid droplet accumulation to osteoarthritis pathogenesis. For instance, Park *et al*. reported that Acot12 knockout reduced chondrocyte cellularity, accelerated cartilage degradation, and exacerbated osteoarthritis progression *via* excessive lipogenesis and lipid droplet accumulation-driven metabolic stress (16), whereas Lee *et al*. demonstrated that Pkck2/Stamp2/Fsp27-induced lipid droplet deposition mitigated lipotoxicity in chondrocytes (17), suggesting context-dependent roles of lipid droplets in osteoarthritis pathology.

**Figure 10:**
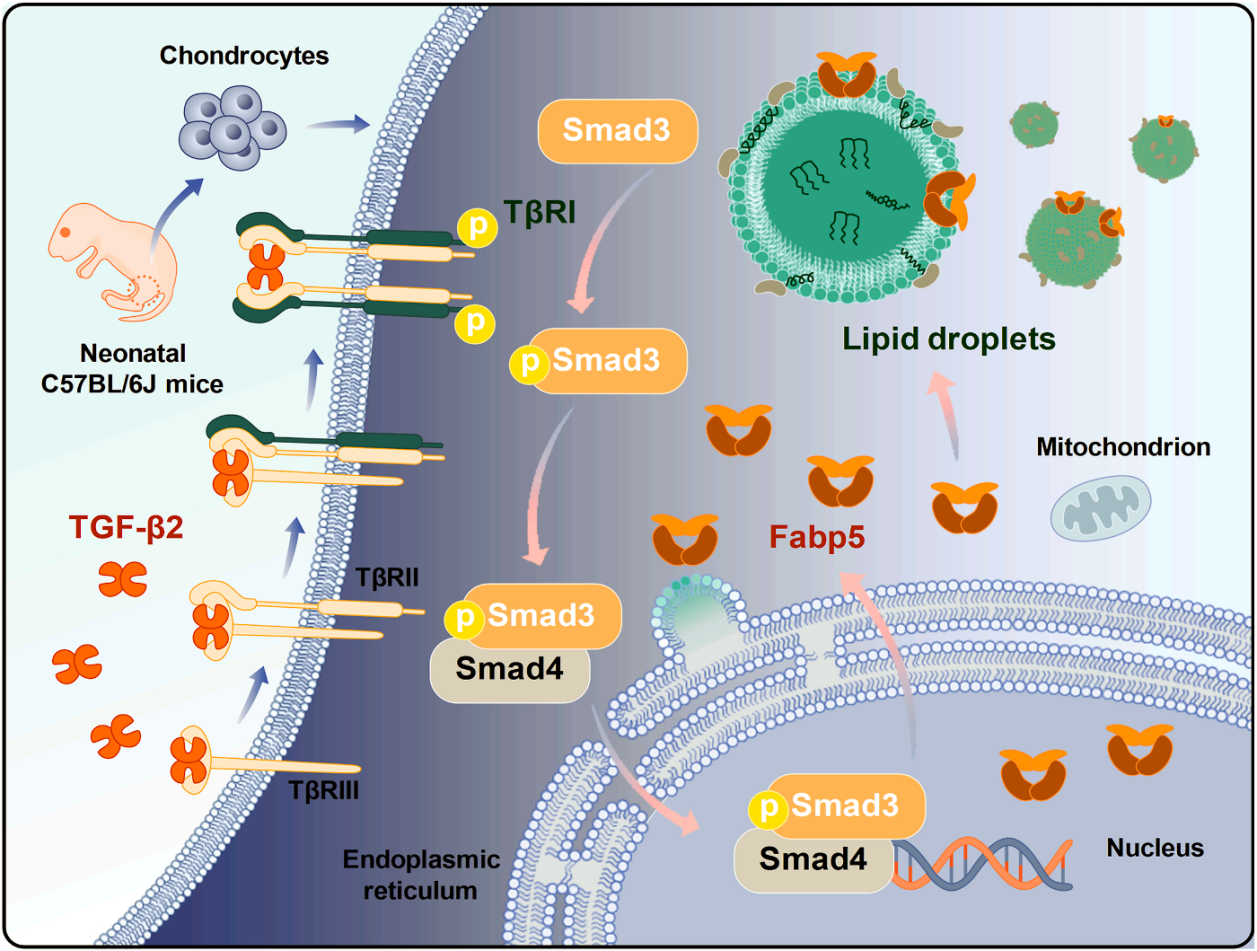
**Schematic path of TGF-β2-induced lipid droplet accumulation in chondrocytes.** Upon binding to TβRI, TGF-β2 triggers the p-Smad3 signaling cascade, leading to the upregulation of the key lipid chaperone Fabp5 and the subsequent accumulation of neutral lipids and lipid droplets in chondrocytes.

Concomitant with osteoarthritis progression, cytokine dysregulation remodels the osteochondral microenvironment (55, 56). Notably, TGF-β2 is triggered and expressed within, adjacent to, and remote from osteoarthritic lesions (57), and its expression escalates particularly in deeper cartilage layers and a greater number of chondrocytes as osteoarthritis progresses (28, 29). Here, we identified TGF-β2 as a potent driver of chondrocyte lipid retention. We revealed that TGF-β2 triggered chondrocyte transcriptional reprogramming, with nearly half of the differentially expressed genes linked to lipid metabolism pathways, including fatty acid metabolism, sphingolipid metabolism, ether lipid metabolism, steroidogenesis, biosynthesis of unsaturated fatty acids, glycosphingolipid biosynthesis (lacto and neolacto series), phospholipase D signaling, and PPAR signaling (Fig. 1, *A–C*) and promoted the neutral lipid and lipid droplet accumulation in chondrocytes *in vitro* (Figs. 1, *D–H*, S1 and S2, Videos S1 and S2), cartilage layers *ex vivo* (Fig. 1, *I* and *J*) and osteoarthritic cartilage *in vivo* (Fig. S3). We demonstrated the regulatory role of Fabp5 (Figs. 2, 3 and S4–S9) and the importance of TβRI/p-Smad3 axis (Figure 4, Figure 5, Figure 6, Figure 7, Figure 8 and S10–S18) in TGF-β2-induced lipid droplet formation. We profiled lipid metabolites (Fig. 9), and extended our analysis to non-lipid metabolic pathway metabolites (Fig. S19) to gain further insight into the broader metabolic landscape in chondrocytes induced by TGF-β2. This modulation process (Fig. 10) contributes to a deeper understanding of metabolic regulation in cartilage triggered by TGF-β2 as a novel lipid rheostat, which is beyond its canonical roles in regulating matrix remodeling.

Fabp5 is an intracellular lipid chaperone with a unique structural signature, a conserved Cys120-Cys127 disulfide bridge formed by six cysteine residues, which confers protein stability through thiol-disulfide exchange and thereby amplifies its functional impacts across physiological and pathological contexts (58). With deeper understanding of its biological role, multiple functions have been reported including but not limited to canonical lipid transport, lipid synthesis and breakdown, and lipid droplet storage. For lipid transport, Fabp5 promotes the lipid transportation to the specific intracellular organelles and achieves biological processes such as lipid storage, trafficking, renewal of membrane components, and lipid-triggered transcriptional regulation in the nuclei (59, 60, 61). Importantly, in Fabp5-dominant cells, Fabp5 exerts a clear pro-trafficking function by escorting retinoic acid from the cytosol to nuclear PPARβ/δ, whereas this route is functionally attenuated in cells with a high CRABP-II/Fabp5 ratio, in which retinoic acid is preferentially redirected to RAR (62). Moreover, unsaturated long-chain fatty acids further promote Fabp5-dependent PPARβ/δ signaling, whereas saturated fatty acids suppress the Fabp5-PPARβ/δ axis by displacing retinoic acid and biasing signaling toward RAR (63). Mechanistically, structural studies showed that linoleic acid and arachidonic acid induce an allosteric rearrangement that exposes a tertiary nuclear localization signal and thereby promotes Fabp5 nuclear translocation, whereas more saturated non-activating fatty acids destabilize this activation loop and thus inhibit productive nuclear trafficking (64). In activated CD8^+^ T cells, Fabp5 supports fatty-acid import, promotes intracellular lipid trafficking, and enhances lipid-fueled mitochondrial respiration, and this process is further reinforced by TAGLN2-mediated cell surface localization of Fabp5 (65). For lipid synthesis, Fabp5 is well recognized to participate in lipid synthesis through regulating fatty acid metabolic pathways, but this biological process is shown to be cell-type dependent. In cervical cancer, Fabp5 expression is positively correlated with the expressions of fatty acid synthetic enzymes FASN and ACC1 (66). Fabp5 overexpression promotes lipolysis and *de novo* fatty acid synthesis, increases the release of intracellular free fatty acids, providing a prerequisite for the lipid droplet formation (67). A liver cancer study shows that E3-ubiquitin ligase TRIM45 can catalyze the ubiquitination of Fabp5 and promote its nuclear translocation. Nuclear Fabp5 interacts with PPARγ, thereby enhancing the expression of downstream lipid synthesis-related genes and forming a TRIM45-Fabp5-PPARγ-fatty acid axis (68). Research on pancreatic neuroendocrine tumors shows that Fabp5 promotes lipid metabolism reprogramming and lipid droplet deposition *via* regulating FASN and activating the Wnt/β-catenin signaling (69). Therefore, the role of Fabp5 in lipid synthesis should be interpreted as its broad involvement in a range of activities from lipid metabolism reprogramming to lipid storage regulation. For lipid breakdown, Fabp5 displays bidirectional, cell-context-dependent effects rather than a uniform pro-catabolic function. In the brain, Fabp5 promotes the intracellular disposition and turnover of endocannabinoid-related lipids by facilitating anandamide hydrolysis to arachidonic acid and then shuttling arachidonic acid to nuclear PPARβ/δ, thereby coupling lipid breakdown to downstream transcriptional signaling (70). In cervical cancer cells, Fabp5 silencing leads to the accumulation of intracellular neutral lipids, while Fabp5 overexpression reduces neutral lipids and increases free fatty acids and glycerol, indicating that Fabp5 can promote lipid droplet lipolysis (66). By contrast, in macrophages, endogenous Fabp5 exerts an inhibitory effect on oxidative lipid breakdown and ATP production by inhibiting the PPARγ signaling (71). Collectively, these data indicate that Fabp5 cannot be simply defined as a factor that universally promotes droplet breakdown (72). For lipid droplet formation, Fabp5 is reported to be involved in lipid storage, lipid droplet deposition, and lipid droplet-related metabolic regulation after fatty acid uptake. In liver cancer cells, oleic acid treatment and hypoxia can activate the Fabp5-HIF-1α axis. Both Fabp5 and HIF-1α silencing can reduce the number of lipid droplets and decrease the expression of lipid storage- and lipid droplet formation-related genes such as ACSL1, GPAT, LIPIN1, and DGAT2. At the same time, CPT1A and ATGL are upregulated, indicating that the Fabp5-HIF-1α axis promotes the storage of fatty acids in the form of lipid droplets (72). A later study on pleural mesothelioma shows that Fabp5 silencing reduces the abundance of BODIPY-labeled lipid droplets and downregulates the expression of phospholipid biosynthesis-related genes such as CCTα, CHPT1, and CEPT1, supporting Fabp5-regulated tumor cell lipid droplet accumulation and lipid metabolism (73). In immune cells, Fabp5 activation can reduce β-oxidation levels, leading to the accumulation of lipid droplets in monocytes, suggesting that Fabp5 can also increase lipid droplets in immune cells by inhibiting lipid consumption (42). Furthermore, Fabp5 can interact with Sept11 and Cav1 and colocalize on the surface of lipid droplets, thus promoting lipid storage and lipid droplet accumulation (40). Similarly, Fabp5 interacts with Rab34 on the surface of lipid droplets to enhance the lipid droplet formation in 3T3-L1 cells (43). In the current study, we showed Fabp5 could promote lipid synthesis and lipid droplet deposition in chondrocytes induced by TGF-β2, with increased colocalization on the surface of lipid droplets (Figs. 2 and 3). These results collectively demonstrate the functional diversity of Fabp5 on lipid regulation from lipid transport to lipid droplet formation and deposition.

The regulatory effects of TGF-β2 on lipid droplet accumulation diverge markedly from other TGF-β superfamily members. For instance, Wang *et al*. reported that TGF-β1 suppressed lipid droplet accumulation in AML12 mouse hepatocytes and liver tissues by downregulating cholesterol biosynthesis enzymes and reducing total cholesterol content (74). Romero-Córdoba *et al*. found that TGF-β1 synergized with calcitriol to trigger lipid droplet accumulation by enhancing fatty acid anabolism and suppressing lipid mobilization in human trophoblasts (75). According to Wang *et al*., GDF11 effectively reduced lipid droplet deposition in condylar chondrocytes and alleviated temporomandibular joint osteoarthritis by downregulating C/EBPα, Fabp4, perilipin1, and adiponectin (76). Additionally, Frohlich *et al*. demonstrated that GDF11 triggered lipid droplet accumulation in hepatocellular carcinoma cells by acutely inducing lipogenic genes and elevating triacylglycerol pools, whereas primary hepatocytes remained refractory to this stimulus (77). This functional pleiotropy likely arises from heterogeneity within the TGF-β superfamily, including divergent transcriptional regulation mechanisms, protein structure features, precursor activation modes, receptor affinity profiles, signal transduction pathways, feedback regulation loops, and spatiotemporal expression patterns (29). Notably, responses across *in vitro*, *ex vivo*, and *in vivo* systems are further modulated by experimental variables such as TGF-β concentration, treatment duration, and cell type-specific signaling thresholds (78). In this study, the concentrations of TGF-β2 (5 and 10 ng/ml) were selected based on two lines of evidence. First, our previous chondrocyte studies showed that these doses profoundly affected cell viability, migration, and proliferation (35) and effectively activated Smad3 signaling (79). Second, this concentration range is consistent with the active TGF-β levels reported in osteoarthritic joints (80, 81, 82, 83). Considering the similar effects of TGF-β2 on lipids and lipid regulators observed in chondrocytes at both 5 and 10 ng/ml, we here selected 5 ng/ml as the optimal concentration and proved that TGF-β2 could effectively promote chondrocyte lipid droplet accumulation through the TβRI/p-Smad3/Fabp5 axis.

TGF-β signaling initiates when the ligand engages with specific transmembrane receptors (TβRI, TβRII, and TβRIII) (29). Active TGF-β2 typically forms a transient complex with TβRIII to recruit TβRII and TβRI; subsequent TβRIII dissociation enables TGF-β2/TβRI/TβRII complex assembly and downstream signal transduction (84). Among these receptors, TβRI, the primary transducer of TGF-β signaling, is indispensable for diverse chondrocyte physiological and pathological processes (85). Matsunaga *et al*. mapped TβRI expression across the entire rat epiphyseal growth plate—from resting to hypertrophic zones—and showed that this receptor drove longitudinal bone elongation through endochondral ossification (86). Sanz-Ramos *et al*. identified TβRI as a critical chondrocyte mechanosensor and noted that TβRI overexpression induced phenotype loss in rat chondrocytes (87). Wang *et al*. demonstrated that postnatal cartilage-specific TβRI knockout induced chondrocyte apoptosis, elevated catabolic factors, suppressed anabolic markers, and precipitated osteoarthritis-like degenerative cartilage lesions (88). In this study, we demonstrated that TβRI inhibition *via* SB525334 downregulated the lipid chaperone Fabp5, thereby attenuating neutral lipid and lipid droplet accumulation in chondrocytes (Figs. 4 and S10–S12). This aligns with the study that TβRI mediated GDF11-induced lipogenesis and lipid droplet accumulation in hepatocellular carcinoma cells (77), yet contrasts with the report that TβRI relayed GDF11 signals to suppress adipogenesis and lipid droplet accumulation in human bone marrow-derived mesenchymal stem cells and 3T3-L1 preadipocytes (89). Such ligand- and cell type-dependent duality underscores the functional complexity of TβRI signaling in lipid droplet accumulation. These findings highlight the necessity of context-specific TβRI targeting to address lipid dysregulation in osteoarthritic cartilage. Therapeutic strategies targeting TβRI must account for ligand identity, cellular microenvironment, and disease stage to avoid paradoxical outcomes.

TGF-β isoforms signal *via* both canonical (Smads) and alternative pathways (*e*.*g*., PI3K/AKT, p38 MAPK, MEK/ERK, and JNK pathways) (90, 91). Smads function as molecular hubs that integrate TGF-β inputs with diverse intracellular networks (92). Our previous work established that TGF-β2 activated p-Smad3 to coordinate chondrocyte behaviors, such as augmenting nanoscale cortical stiffness through cytoskeleton-focal adhesion plaque condensation (93), enhancing connexin 43-mediated gap junction communication (35), and elevating glycolytic flux *via* 6-phosphofructokinase upregulation (79). Here, we extend these findings by demonstrating that TGF-β2/p-Smad3 signaling drove Fabp5-mediated lipid droplet accumulation in chondrocytes (Figure 5, Figure 6 and S13–S17). This mechanism aligns with previous studies linking p-Smad3 to lipid droplet accumulation. For instance, Gu *et al*. revealed that inhibition of TGF-β1/p-Smad3 signaling by pterostilbene suppressed TNF receptor superfamily member 6 and sterol regulatory element-binding protein 1 and ultimately alleviated ectopic lipid droplet accumulation in kidneys of high-fat diet-induced diabetic mice (94). Huang *et al*. reported that inhibition of p-Smad3 reduced lipid droplet accumulation *via* NDRG1-dependent fatty acid oxidation in kidneys of cisplatin-induced acute kidney injury mice (95). Conversely, Luo *et al*. demonstrated that GDF11-activated Smad2/3 reduced lipid droplet accumulation and inhibited adipogenesis differentiation in both human bone marrow-derived mesenchymal stem cells and 3T3-L1 preadipocytes (89). Ouyang *et al*. revealed more complex Smad changes underlying adipogenic gene induction and lipid droplet expansion in 3T3-L1 preadipocytes induced by miR-181a-5p. Specifically, miR-181a-5p-mediated Smad7 suppression promoted Smad3 phosphorylation but reduced Smad4 expression, ultimately attenuating the nuclear translocation of the TGF-β signaling complex (96). These context-dependent outcomes driven by cellular specialization, ligand identity, and microenvironmental cues highlight the challenges of targeting TGF-β2-induced lipid dysregulation in osteoarthritis. Future studies have to dissect spatiotemporal Smad activity and its crosstalk with non-Smad pathways to prevent lipid-driven metabolic stress in chondrocytes and balance metabolic homeostasis in cartilage. In the current study, we provided evidence of a correlation between Smad3 and Fabp5 to indicate the importance of Smads in lipid deposition and droplet formation. ChIP-qPCR provided solid evidence linking p-Smad3 to the transcriptional regulation of Fabp5 by demonstrating p-Smad3 enrichment at an SBE-like region of the Fabp5 promoter region (Fig. 7). These findings support promoter occupancy but do not establish which predicted cis-elements within this region are functionally required for transcriptional activation. Future promoter luciferase and site-directed mutagenesis assays will therefore be necessary to define the precise Smad3-responsive sequence(s).

The mechanisms by which TβRs couple extracellular ligand binding to intracellular Smad activation remain incompletely defined. We here demonstrate that TGF-β2-TβRI engagement selectively phosphorylated Smad3 in chondrocytes (Figs. 8 and S18). Consistently, Jeong *et al*. reported that TβRI inhibition attenuated p-Smad3 signaling and restored muscle regeneration in muscle stem cells of acute experimental diabetic mice (97). Similarly, Dou *et al*. demonstrated that Smad3-TβRI interaction blockade *via* chrysophanol suppressed p-Smad3 expression and ameliorated renal fibrosis in unilateral ureteral obstruction mice (98). Canonically, TGF-β binding triggers TβRII-mediated TβRI phosphorylation, enabling TβRI-dependent heteromeric receptor complexes to phosphorylate Smad C-terminal domains and propagate downstream signaling (99). However, TGF-β signaling fidelity is modulated by receptor endocytic routes: clathrin-mediated TβRI endocytosis promotes Smad2/3 phosphorylation, whereas caveolae-dependent internalization drives receptor degradation and non-Smad pathway dominance (100). Further complexity arises from post-translational modifications (*e*.*g*., glycosylation, ubiquitylation, neddylation, and sumoylation) of TβRs that fine-tune signal duration and specificity (100). Collectively, these regulatory layers underscore the need to delineate spatiotemporal dynamics of TβRI-mediated p-Smad3 activation in chondrocytes under TGF-β2 stimulation, particularly in the context of osteoarthritis-associated lipid metabolic dysregulation.

Notably, healthy chondrocytes rely on the homeostasis of intracellular metabolic activities broadly involving carbohydrate, lipid, amino acid, and nucleotide metabolism (101). During osteoarthritic acute inflammation phase and subsequent degenerative process, elevated levels of TGF-β2 appear in the joint cavity (29, 35, 79). Here we demonstrated the effect of TGF-β2 on the lipid metabolism of chondrocytes, providing new insights into the pathogenesis of cartilage metabolism and osteoarthritis progression. Perturbation of lipid metabolism triggered by growth factors such as TGF-β2 is important for chondrocytes because imbalanced lipid metabolism leads to obstruction of membrane structure renewal, as well as changes in mitochondrial oxidative phosphorylation and reduced ATP production due to the insufficient supply of lipid raw materials (102, 103). It is because of the balanced lipid metabolism that other metabolic activities, such as amino acid metabolism, carbohydrate metabolism, nucleic acid metabolism, vitamin metabolism, *etc*., are ensured to be in a steady state, and the cell behaviors including cell viability, proliferation, migration and differentiation are to be normal. In addition to the marked alterations in lipid metabolism, our wide-targeted metabolomic analysis indicated that TGF-β2 induced a broader metabolic reprogramming in chondrocytes, involving carbohydrate, amino acid, and nucleotide metabolism (Figs. 9 and S19). In terms of carbohydrate metabolism, our previous study showed that TGF-β2 promoted glycolysis in chondrocytes (79). Altered carbohydrate metabolism may provide reducing equivalents required for fatty acid synthesis through the pentose phosphate pathway (104), as well as glycerol-3-phosphate through glycolysis to support glycerophospholipid and triacylglycerol synthesis (105). Changes in amino acid metabolism, particularly glutamine metabolism, may contribute to anaplerotic flux and biosynthetic intermediates that sustain lipogenesis (106). Additionally, alterations of nucleotide-related metabolites may reflect a broader anabolic demand and adaptive metabolic reprogramming (107). Collectively, these data suggest that TGF-β2 affects multiple aspects of chondrocyte metabolism and represents a versatile star factor, underscoring its complexity in osteoarthritis pathogenesis.

Lipid droplets are increasingly recognized as more than just inert lipid containers. Their biological significance lies in their ability to sequester excess fatty acids as neutral lipids and thus limit lipotoxic stress; meanwhile, their mobilization provides substrates for energy production and membrane synthesis. Through these biological functions, lipid droplet dynamics can influence cell behaviors including proliferation, differentiation, and migration. For example, lipid droplet accumulation and catabolism are tightly coupled to metabolic signaling that supports proliferation of liver cancer cells (108). In neural stem/progenitor cells, higher lipid droplet content is linked to greater proliferative capacity due to increased ATGL-mediated access to triacylglycerols, and a greater number of asymmetrically inherited lipid droplets predict earlier cell division; in the same system, experimentally increasing lipid droplets promotes neuronal differentiation at least partly at the expense of astrocyte generation (109). In enterocytes, a switch from lipid droplet assembly to chylomicron biosynthesis is associated with enterocyte migration (110). In oral squamous cell carcinoma cells, perilipin 2-dependent lipid droplet accumulation enhances migration and invasion by activating the PI3K/AKT/mTOR pathway and inducing epithelial-mesenchymal transition (111). As for chondrocytes, our previous study showed that TGF-β2 promoted chondrocyte migration by scratch wound assay and enhanced chondrocyte proliferation by CCK-8 assay (35). These cellular behavior changes might be potentially correlated with cellular lipid metabolism. In particular, TGF-β2-induced lipid droplet accumulation may be beneficial for healthy chondrocytes by buffering lipotoxic stress and sustaining cellular energy homeostasis, thereby supporting chondrocyte survival and functioning (112). The normal lipid metabolism and following ATP generation may, at least in part, explain the pro-proliferative, pro-differentiation, and pro-migratory effects of TGF-β2. In contrast, in osteoarthritic chondrocytes, where mitochondrial function and metabolic flexibility are already compromised, further lipid droplet accumulation induced by TGF-β2 may become detrimental by impairing lipid turnover, exacerbating lipotoxic stress, and promoting chondrocyte dysfunction and death (113). However, the present study does not directly demonstrate the effects of TGF-β2-driven lipid droplet accumulation on chondrocyte behaviors. Future studies combining functional assays with pharmacological or genetic modulation of lipid droplet turnover would be necessary to show whether lipid droplets mechanistically mediate cell fate.

## Experimental procedure

### Chondrocyte isolation and culture

All animal protocols were approved by the Institutional Review Board at the West China Hospital of Stomatology (No. WCHSIRB-D-2023-152) prior to experimentations. Chondrocytes were obtained from the articular cartilage tissues of neonatal C57BL/6J mice (1–3 days) following established guidelines (50, 114). In short, collected cartilage pieces were enzymatically dissociated and the chondrocyte suspension was centrifuged (1000 r/min, 5 min). The pelleted chondrocytes were resuspended in complete media for growth. Chondrocytes were used at passages 1 to 2.

### Organ culture of metatarsals

Metatarsal tissues from neonatal C57BL/6J mice (1–3 days) were collected and cultured *ex vivo* as described (115). In short, under a dissection microscope, the superficial tissues on the tarsals were snipped, and two longitudinal incisions were made toward the phalanges to remove the foot skin and fully expose the metatarsals. The connective tissues between all five metatarsals or phalanges were severed with forceps. After discarding the two outermost tarsal-adjacent metatarsals and excising the phalanges, the central three metatarsals were isolated and cultured for 10 days with/without the treatment of TGF-β2 (100-35B, Peprotech, Cranbury, NJ, USA) (5 ng/ml). After fixation and dehydration, metatarsals were embedded in Epredia Neg-50 Frozen Section Medium (22-110-617, Thermo Fisher Scientific) and processed into 5-μm-thick frozen sections.

### Knee osteoarthritic mouse model establishment by anterior cruciate ligament transection (ACLT)

C57BL/6J mice (8 weeks old, n = 34 per sex) were anesthetized and subjected to unilateral ACLT surgery to induce post-traumatic osteoarthritis, as previously described (5). Briefly, the right knee joint underwent lateral arthrotomy, the patella was displaced medially, and the knee was fully flexed. The anterior cruciate ligament was then clearly exposed and transected under direct visualization to induce joint instability. The contralateral left knee underwent sham arthrotomy with the ligament preserved. After surgery, mice received postoperative analgesia and were allowed unrestricted cage activity. This model was selected because ACLT is a well-established surgical model of post-traumatic osteoarthritis in which ACLT produces immediate cartilage microinjury as well as persistent joint instability, thereby leading to abnormal mechanical loading, catabolic joint responses, progressive cartilage degeneration, and osteophyte/structural joint changes (116). At 8 weeks after ACLT surgery, hindlimbs were harvested, fixed, decalcified, embedded in paraffin, and sectioned at 5 μm for histological evaluation. We here chose to harvest knee joint samples at 8 weeks post-surgery, because 8-week period represents an established late-stage/advanced stage characterized by substantial cartilage degeneration and structural joint changes, whereas 4-week period was generally considered an early stage of experimental post-traumatic osteoarthritis (117). Therefore, the cartilage tissues analyzed in the present study exhibited more typical characteristics of osteoarthritic cartilage and the results could better reflect the actual changes in the progression of osteoarthritis.

### Bulk RNA sequencing

Total RNA from articular chondrocytes from neonatal C57BL/6J mice induced by TGF-β2 (5 ng/ml, 24 h) was extracted with Trizol (15596018CN, Thermo Fisher Scientific) and outsourced for library construction and sequencing (Lifegenes Biotechnology, Shanghai, CN). After quality filtering of raw fastq reads with in-house Perl scripts and genomic alignment, transcript levels were calculated using the FPKM method. Differential expression was determined with a |log_2_(fold change)| > 0.585 (fold change > 1.5) and an adjusted *p*-value < 0.05. The functional profiling of these genes was carried out *via* GO and KEGG enrichment analyses.

### Metabolomics

Metabolomic profiling was performed on primary chondrocytes isolated from neonatal C57BL/6J mice after TGF-β2 treatment (5 ng/ml, 48 h) as described (118). Cells were harvested on ice by scraping, centrifuged at 1000*g* for 10 min, snap-frozen in liquid nitrogen for 15 min, and sent to Metware Biotechnology for LC-ESI-MS/MS analysis. For extraction, samples were thawed on ice, mixed with 1 ml pre-cooled 80% methanol, vortexed for 2 min, and subjected to three freeze-thaw cycles. After centrifugation at 12,000 r/min for 10 min at 4 °C, 200 μl of supernatant was transferred for analysis. Four biological replicates were included per group.

Metabolites were analyzed on an ExionLC AD UPLC system coupled to a QTRAP LC-MS/MS system equipped with an ESI Turbo Ion-Spray interface and controlled by Analyst 1.6.3 software (Sciex). Chromatographic separation was performed using both a Waters ACQUITY UPLC HSS T3 C18 column and a Waters ACQUITY UPLC BEH Amide column at 40 °C with a flow rate of 0.4 ml/min and an injection volume of 2 μl. Data were acquired in both positive and negative ion modes using scheduled MRM.

PCA was performed in R using the prcomp function after unit-variance scaling. Hierarchical clustering and Pearson correlation analysis were conducted using the ComplexHeatmap package. OPLS-DA was performed with MetaboAnalystR after log_2_ transformation and mean-centering, and model overfitting was assessed by 200-permutation testing. Differential metabolites were defined as those with VIP ≥ 1 and |log_2_(fold change)| > 1 (fold change > 2.0). KEGG-based pathway enrichment analysis was performed using a hypergeometric test.

### siRNA transfection

Chondrocytes cultured in antibiotic-free complete media were transfected with 50 nM siRNA for 24 h. The siRNA sequences, provided by Hanbio, were listed below: for si-NC, the sense and antisense sequences were 5′-UUCUCCGAACGUGUCACGU-3′ and 5′-ACGUGACACGUUCGGAGAA-3′, respectively; for si-Fabp5-1, the sense and antisense sequences were 5′-GCAACAACAUCACGGUCAA-3′ and 5′-UUGACCGUGAUGUUGUUGC-3′, respectively; for si-Fabp5-2, the sense and antisense sequences were 5′-CGACUGUGUUCUCUUGUAA-3′ and 5′-UUACAAGAGAACACAGUCG-3′, respectively; for si-Fabp5-3, the sense and antisense sequences were 5′-GAGAAGUUUGAUGAAACGACA-3′ and 5′-UCGUUUCAUCAAACUUCUCUC-3′, respectively; for si-TGF-β2-1, the sense and antisense sequences were 5′-CGAUAGCAAGGUUGUGAAA-3′ and 5′-UUUCACAACCUUGCUAUCG-3′, respectively; for si-TGF-β2-2, the sense and antisense sequences were 5′-GGAGCGACGAGGAGUACUA-3′ and 5′-UAGUACUCCUCGUCGCUCC-3′, respectively; and for si-TGF-β2-3, the sense and antisense sequences were 5′-CGCUUUGAUGUCUCAACAA-3′ and 5′-UUGUUGAGACAUCAAAGCG-3′, respectively.

### Quantitative real-time polymerase chain reaction (qPCR)

siRNA-mediated gene knockdown in chondrocytes was confirmed through qPCR following established protocols (114). Total RNA was isolated using a spin column-based method to ensure integrity and purity. The qPCR template (cDNA) was generated from spectrophotometrically-qualified RNA by reverse transcription. The qPCR primer pairs were designed to specifically amplify hypoxanthine-guanine phosphoribosyltransferase (Hprt; F: 5′-TTGGGCTTACCTCACTGCTT-3′, R: 5′-GCAAAAAGCGGTCTGAGGAG-3′), Fabp5 (F: 5′-TGAAAGAGCTAGGAGTAGGACTG-3′, R: 5′-CTCTCGGTTTTGACCGTGATG-3′), and TGF-β2 (F: 5′-CTTCGACGTGACAGACGCT-3′, R: 5′-GCAGGGGCAGTGTAAACTTATT-3′). qPCR was performed using a CFX96™ Real-Time System (Bio-Rad, Hercules, CA, USA). Gene expression was analyzed using the 2^-ΔΔCq^ algorithm with normalization to Hprt.

### Western blotting

Protein analysis by Western blotting followed our established protocol (115, 116). Chondrocytes were stimulated with 5 or 10 ng/ml TGF-β2 for durations spanning 15 min to 72 h. In pharmacological inhibition studies, chondrocytes received 12-h pretreatment with 10 μm SB525334 (prepared in DMSO; HY-12043, MedChemExpress) or 6-h pretreatment with 5 μm SIS3 (prepared in DMSO; 566,405, Sigma-Aldrich, St Louis, MO, USA). Total proteins in chondrocyte lysates were resolved by electrophoresis and electrotransferred onto PVDF membranes (IPVH00010, Millipore). After an incubation with specific primary antibodies (1:1,000, 12 h, 4 °C) against β-actin (sc-47778, Santa Cruz Biotechnology), Fabp5 (39,926, Cell Signaling Technology), phosphorylated Smad3 (p-Smad3; R22919, Zen-Bioscience), Smad3 (ab208182, Abcam), and Smad4 (ab40759, Abcam), membranes were treated with species-matched HRP-conjugated secondary antibodies (sc-2357 or sc-516102, Santa Cruz Biotechnology; 1:5,000, 2 h, room temperature). Antibody specificity was supported by the manufacturer’s datasheets and previous publications. Western blot bands were quantified by densitometry using ImageJ/Fiji (NIH, Bethesda, MD, USA) after background subtraction. Signals were normalized to β-actin and expressed relative to controls.

### Immunofluorescence

Immunofluorescence procedures were performed as previously described (5, 114). Fixed and permeabilized samples were blocked for 1 h under specific conditions: chondrocytes with 5% BSA (A1933, Sigma-Aldrich); frozen sections with 10% normal goat serum (SL038, Solarbio Life Sciences); and paraffin-embedded sections, which were first deparaffinized in xylene and rehydrated through an ethanol series, with 10% normal goat serum. Samples were probed with primary antibodies against Fabp5 (506,373, Zen-Bioscience), p-Smad3 (R22919, Zen-Bioscience), and Osx (ab22552, Abcam) (1:200, 12 h, 4 °C), followed by Alexa Fluor-conjugated secondary antibodies (A-31573 or A-11008, Life Technologies; 1:200, 2 h, room temperature). Antibody specificity was supported by the manufacturer’s datasheets and previous publications. Additional staining included: cytoskeletal visualization with FITC- or TRITC phalloidin (A12379 or R415, Invitrogen, Carlsbad, CA, USA; 1:40, 12 h, 4 °C), nuclear counterstaining with Dapi (D9542, Sigma-Aldrich; 1:100, 10 min, room temperature), and neutral lipid labeling with BODIPY 493/503 (D3922, Thermo Fisher Scientific; 10 min, room temperature). Imaging was performed on a FV3000 confocal laser scanning microscope (Olympus, Tokyo, JP) for chondrocytes, and a VS200 research slide scanner (Olympus) for cartilage tissues. Immunofluorescence images were analyzed using ImageJ/Fiji software. Linear and mean fluorescence intensity were measured for regions of interest after background subtraction. Colocalization was quantified as Manders’ coefficients. Lipid droplet number per cell was determined by defining single-cell ROIs, thresholding the lipid droplet signal, converting to a binary mask, and counting droplets using Analyze Particles.

### Bioinformatic prediction of Smad3-binding motifs in the promoter region of Fabp5

The mouse Fabp5 promoter sequence, −2000 to +200 bp at the TSS, was retrieved from the National Center for Biotechnology Information (https://www.ncbi.nlm.nih.gov/gene/?term=Fabp5). Promoter binding prediction was performed with vertebrate JASPAR Smad3 matrix (https://jaspar.elixir.no/matrix/MA0795.1/).

### ChIP-qPCR

ChIP-qPCR was performed as previously described (116, 119). Briefly, chromatin was cross-linked with 1% formaldehyde, digested with micrococcal nuclease, and immunoprecipitated using the Pierce Magnetic ChIP Kit (26,157, Thermo Fisher Scientific). ChIP-qPCR was performed in chondrocytes using a ChIP-grade p-Smad3 antibody (9520, Cell Signaling Technology), primer sets flanking the SBE-like candidate site in the Fabp5 promoter region (F: 5′-GTAATCTCCCTTAGAGACTGCTGCC-3′, R: 5′-TCACTGGGGCCTCTTGGTT-3′), and primer sets flanking the non-canonical/GC-rich candidate region in the Fabp5 promoter region (F: 5′-GTGATGGTCAGGAGCTACTA-3′, R: 5′-TTGAGTAAAGGTTATGGAGCG-3′). A negative control region lacking predicted Smad3-binding motifs was set using Fabp5_NegCtrl primers (F: 5′-GATAACTTCCTCAGGAATCGC-3′, R: 5′-CTATCACCACGGAGACAAGG-3′). ChIP enrichment was presented relative to the IgG control.

### Biochemical determination of triglycerides and cholesteryl esters

Neutral lipids were extracted in isopropanol and centrifuged at approximately 12,000*g* for 5 min. The collected supernatants were diluted five-fold with assay buffer to ensure that the isopropanol content did not exceed 20%. Triglyceride contents were measured by using the Amplex Red Triglyceride Assay Kit (S0219, Beyotime, Shanghai, CN) and cholesteryl ester contents were measured by using the Amplex Red Cholesterol and Cholesteryl Ester Assay Kit (S0211, Beyotime). Signals were measured and analyzed by absorbance at 570 nm.

### Statistics

Experimental data (n ≥ 3) were reported as mean ± SD. Statistical analysis was performed using unpaired two-tailed Student’s *t* test for two groups, and ANOVA followed by Tukey’s honestly significant difference (HSD) *post hoc* test for multiple groups. The threshold for statistical significance was set at 0.05.

## Data availability

All data are available from the corresponding author upon reasonable request.

## Supporting information

This article contains supporting information.

## Conflict of interest

The authors declare that they have no conflicts of interest with the contents of this article.

## Supporting information

Supplementary Material

Supplementary Videos
