# Supplementary material for: TGF-β2 drives lipid droplet accumulation in chondrocytes through the TβRI/p-smad3/fabp5 axis
Source: J Biol Chem. 2026 Jul 6;302(8):113317. doi: 10.1016/j.jbc.2026.113317 (PMC13453483; doi:10.1016/j.jbc.2026.113317)
Supplement: Supplementary file 2 [file mmc2.doc]

Supplementary materials

**TGF-β2 drives lipid droplet accumulation in chondrocytes through the TβRI/p-Smad3/Fabp5 axis**

Jieya Wei, Caixia Pi, Yu Qi, Wenbin Yang, Jianxun Sun, Xin Xu, Jing Xie

**Supplementary figures**

**Figure S1**

**Figure S1. TGF‐β2 induces lipid droplet accumulation in a collective of chondrocytes.**

Representative immunofluorescence micrographs demonstrating a significant increase in neutral lipids and lipid droplets in a collective of chondrocytes following a 48-h treatment with 5 ng/ml TGF-β2. Neutral lipids and lipid droplets are visualized with BODIPY 493/503 (green), F-actin cytoskeleton is delineated with TRITC-phalloidin (red), and nuclei are labeled with Dapi (blue). Images are representative of six independent experiments (n = 6). Scale bars: 50 μm (first four columns) and 15 μm (last two columns, enlarged images).

**Figure S2**

**Figure S2. TGF-β2 increases intracellular contents of triglycerides and cholesteryl esters in chondrocytes.**

**(a)** Bar chart showing the change of triglyceride contents in chondrocytes treated with TGF-β2, measured by Beyotime Amplex Red Triglyceride Assay Kit. Data from three independent experiments (n = 3) were analyzed by unpaired two-tailed Student's t-test and are presented as mean ± SD.

**(b)** Bar chart showing the change of cholesteryl ester contents in chondrocytes treated with TGF-β2, measured by Beyotime Amplex Red Cholesterol and Cholesteryl Ester Assay Kit. Data from three independent experiments (n = 3) were analyzed by unpaired two-tailed Student's t-test and are presented as mean ± SD.

**Figure S3**

**Figure S3. Osteoarthritic cartilage exhibits substantial neutral lipid accumulation.**

**(a)** Representative immunofluorescence micrographs showing increased neutral lipid accumulation in the cartilage of knee osteoarthritic mouse models induced by ACLT. Neutral lipids are visualized with BODIPY 493/503 (green). Osx is labeled in red (Alexa Fluor^®^ 647), differentiating mature cartilage (negative) from calcified cartilage (positive). Nuclei are stained with Dapi (blue). Images are representative of three independent experiments (n = 3). Scale bars: 200 μm (first two columns) and 40 μm (last column, enlarged images).

**(b)** Mean fluorescence intensity quantification validating the observed neutral lipid abundance in (a). Data from 6 replicates of three independent experiments (n = 3) were analyzed by unpaired two-tailed Student's t-test and are presented as mean ± SD.

**Figure S4**

**Figure S4. TGF‐β2 induces the upregulation of Fabp5 in a collective of chondrocytes.**

Representative immunofluorescence micrographs showing a substantial increase in Fabp5 expression in the cytoplasm and nuclei of a collective of chondrocytes following a 48-h incubation with 5 ng/ml TGF-β2. Fabp5 is labeled in red (Alexa Fluor^®^ 647). F-actin cytoskeleton is visualized with FITC-phalloidin (green), and nuclei are stained with Dapi (blue). Images are representative of six independent experiments (n = 6). Scale bars: 50 μm (first four columns) and 15 μm (last two columns, enlarged images).

**Figure S5**

**Figure S5.** **Osteoarthritic cartilage shows elevated Fabp5 expression.**

**(a)** Representative immunofluorescence micrographs showing a significant increase in Fabp5 expression in the cartilage of knee osteoarthritic mouse models induced by ACLT. Fabp5 is labeled in red (Alexa Fluor^®^ 647). Osx is labeled in green (Alexa Fluor^®^ 488), differentiating mature cartilage (negative) from calcified cartilage (positive). Nuclei are stained with Dapi (blue). Images are representative of three independent experiments (n = 3). Scale bars: 400 μm (first two columns) and 40 μm (last column, enlarged images).

**(b)** Mean fluorescence intensity quantification validating the Fabp5 abundance in (a). Data from 6 replicates of three independent experiments (n = 3) were analyzed by unpaired two-tailed Student's t-test and are presented as mean ± SD.

**Figure S6**

**Figure S6. si-Fabp5** **transfection reduces Fabp5 fluorescence intensity, neutral lipid accumulation, and lipid droplet abundance in TGF-β2-treated chondrocytes.**

**(a)** Mean fluorescence intensity quantification per chondrocyte confirming that transfection with si-Fabp5-3 at 50 nM significantly decreased Fabp5 expression in chondrocytes in the presence of TGF-β2 at 5 ng/ml for 48 h in **Figure 3d**. Data from 30 individual cell replicates of six independent experiments (n = 6) were analyzed by one-way ANOVA and are presented as mean ± SD.

**(b)** Mean fluorescence intensity quantification per chondrocyte validating that transfection with si-Fabp5-3 at 50 nM significantly decreased neutral lipid abundance in chondrocytes in the presence of TGF-β2 at 5 ng/ml for 48 h in **Figure 3d**. Data from 30 individual cell replicates of six independent experiments (n = 6) were analyzed by one-way ANOVA and are presented as mean ± SD.

**(c)** Quantitative analysis of the lipid droplet number per chondrocyte confirming that transfection with si-Fabp5-3 at 50 nM significantly decreased lipid droplets in chondrocytes in the presence of TGF-β2 at 5 ng/ml for 48 h in **Figure 3d**. Data from 30 individual cell replicates of six independent experiments (n = 6) were analyzed by one-way ANOVA and are presented as mean ± SD.

**Figure S7**

**Figure S7. Fabp5 knockdown decreases intracellular contents of triglycerides and cholesteryl esters in TGF-β2-treated chondrocytes.**

**(a)** Bar chart showing the change of triglyceride contents in chondrocytes transfected with si-Fabp5 in the presence of TGF-β2, measured by Beyotime Amplex Red Triglyceride Assay Kit. Data from three independent experiments (n = 3) were analyzed by one-way ANOVA and are presented as mean ± SD.

**(b)** Bar chart showing the change of cholesteryl ester contents in chondrocytes transfected with si-Fabp5 in the presence of TGF-β2, measured by Beyotime Amplex Red Cholesterol and Cholesteryl Ester Assay Kit. Data from three independent experiments (n = 3) were analyzed by one-way ANOVA and are presented as mean ± SD.

**Figure S8**

**Figure S8. Endogenous TGF-β2 knockdown by si-RNA reduces Fabp5 fluorescence intensity, neutral lipid accumulation, and lipid droplet abundance in chondrocytes.**

**(a)** Mean fluorescence intensity quantification per chondrocyte confirming that transfection with si-TGF-β2-1 at 50 nM significantly decreased Fabp5 expression in chondrocytes in **Figure 3j**. Data from 30 individual cell replicates of six independent experiments (n = 6) were analyzed by unpaired two-tailed Student's t-test and are presented as mean ± SD.

**(b)** Mean fluorescence intensity quantification per chondrocyte validating that transfection with si-TGF-β2-1 at 50 nM significantly reduced neutral lipid accumulation in chondrocytes in **Figure 3j**. Data from 30 individual cell replicates of six independent experiments (n = 6) were analyzed by unpaired two-tailed Student's t-test and are presented as mean ± SD.

**(c)** Quantitative analysis of the lipid droplet number per chondrocyte confirming that transfection with si-TGF-β2-1 at 50 nM significantly reduced lipid droplet accumulation in chondrocytes in **Figure 3j**. Data from 30 individual cell replicates of six independent experiments (n = 6) were analyzed by unpaired two-tailed Student's t-test and are presented as mean ± SD.

**Figure S9**

**Figure S9. Endogenous TGF-β2 knockdown decreases triglyceride and cholesteryl ester contents in chondrocytes.**

**(a)** Bar chart showing the change of triglyceride contents in chondrocytes transfected with si-TGF-β2, measured by Beyotime Amplex Red Triglyceride Assay Kit. Data from three independent experiments (n = 3) were analyzed by unpaired two-tailed Student's t-test and are presented as mean ± SD.

**(b)** Bar chart showing the change of cholesteryl ester contents in chondrocytes transfected with si-TGF-β2, measured by Beyotime Amplex Red Cholesterol and Cholesteryl Ester Assay Kit. Data from three independent experiments (n = 3) were analyzed by unpaired two-tailed Student's t-test and are presented as mean ± SD.

**Figure S10**

**Figure S10.** **Inhibition of TβRI decreases the expression of Fabp5 in a collective of chondrocytes in the presence of TGF‐β2.**

Representative immunofluorescence micrographs showing that inhibition of TβRI by SB525334 suppressed both cytoplasmic and nuclear Fabp5 expression in a collective of chondrocytes in the presence of TGF-β2 (5 ng/ml). Fabp5 is visualized in red (Alexa Fluor^®^ 647), F-actin cytoskeleton is labeled with FITC-phalloidin (green), and nuclei are labeled with Dapi (blue). Images are representative of five independent experiments (n = 5). Scale bars: 50 μm (first four columns) and 15 μm (last two columns, enlarged images).

**Figure S11**

**Figure S11.** **Inhibition of TβRI decreases neutral lipids and lipid droplets in a collective of chondrocytes in the presence of TGF‐β2.**

Representative immunofluorescence micrographs showing that blockade of TβRI by SB525334 attenuated TGF-β2-driven accumulation of neutral lipids and lipid droplets in a collective of chondrocytes. BODIPY 493/503 visualizes neutral lipids and lipid droplets (green), TRITC-phalloidin delineates F-actin cytoskeleton (red), and Dapi labels nuclei (blue). Images are representative of five independent experiments (n = 5). Scale bars: 50 μm (first four columns) and 15 μm (last two columns, enlarged images).

**Figure S12**

**Figure S12. Inhibition of TβRI decreases triglyceride and cholesteryl ester contents in TGF-β2-treated chondrocytes.**

**(a)** Bar chart showing the change of triglyceride contents in chondrocytes following inhibition of TβRI by SB525334 in the presence of TGF-β2, measured by Beyotime Amplex Red Triglyceride Assay Kit. Data from three independent experiments (n = 3) were analyzed by one-way ANOVA and are presented as mean ± SD.

**(b)** Bar chart showing the change of cholesteryl ester contents in chondrocytes following inhibition of TβRI by SB525334 in the presence of TGF-β2, measured by Beyotime Amplex Red Cholesterol and Cholesteryl Ester Assay Kit. Data from three independent experiments (n = 3) were analyzed by one-way ANOVA and are presented as mean ± SD.

**Figure S13**

**Figure S13.** **TGF‐β2 rapidly upregulates p-Smad3 in a collective of chondrocytes.**

Representative immunofluorescence micrographs showing a significant increase in p-Smad3 expression, especially in nuclei of a collective of chondrocytes, after a 15-min incubation with 5 ng/ml TGF-β2. p-Smad3 is labeled in red (Alexa Fluor^®^ 647), F-actin cytoskeleton is visualized with FITC-phalloidin (green), and nuclei are stained with Dapi (blue). Images are representative of six independent experiments (n = 6). Scale bars: 50 μm (first four columns) and 15 μm (last two columns, enlarged images).

**Figure S14**

**Figure S14. The expression of p-Smad3 in a collective of chondrocytes induced by SIS3 in the presence of TGF‐β2.**

Representative immunofluorescence micrographs showing that SIS3 decreased p-Smad3 expression, especially in nuclei of a collective of chondrocytes in the presence of TGF-β2 (5 ng/ml). p-Smad3 is labeled in red (Alexa Fluor^®^ 647), F-actin cytoskeleton is labeled with FITC-phalloidin (green), and nuclei are stained with Dapi (blue). Images are representative of five independent experiments (n = 5). Scale bars: 50 μm (first four columns) and 15 μm (last two columns, enlarged images).

**Figure S15**

**Figure S15. Inhibition of p-Smad3 reduces the expression of Fabp5 in a collective of chondrocytes in the presence of TGF‐β2.**

Representative immunofluorescence micrographs showing that inhibition of p-Smad3 signaling by SIS3 reduced Fabp5 expression in a collective of chondrocytes in the presence of TGF-β2 (5 ng/ml). Fabp5 is labeled in red (Alexa Fluor^®^ 647), F-actin cytoskeleton is labeled with FITC-phalloidin (green), and nuclei are stained with Dapi (blue). Images are representative of five independent experiments (n = 5). Scale bars: 50 μm (first four columns) and 15 μm (last two columns, enlarged images).

**Figure S16**

**Figure S16. Inhibition of** **p-Smad3 reduces TGF‐β2-driven lipid droplet accumulation in a collective of chondrocytes in the presence of TGF‐β2.**

Representative immunofluorescence micrographs showing that blockade of p-Smad3 signaling by SIS3 attenuated TGF-β2-driven accumulation of neutral lipids and lipid droplets in a collective of chondrocytes. BODIPY 493/503 visualizes neutral lipids and lipid droplets (green), TRITC-phalloidin delineates F-actin cytoskeleton (red), and Dapi labels nuclei (blue). Images are representative of five independent experiments (n = 5). Scale bars: 50 μm (first four columns) and 15 μm (last two columns, enlarged images).

**Figure S17**

**Figure S17. Inhibition of p-Smad3 decreases triglyceride and cholesteryl ester contents in TGF-β2-treated chondrocytes.**

**(a)** Bar chart showing the change of triglyceride contents in chondrocytes following inhibition of p-Smad3 by SIS3 in the presence of TGF-β2, measured by Beyotime Amplex Red Triglyceride Assay Kit. Data from three independent experiments (n = 3) were analyzed by one-way ANOVA and are presented as mean ± SD.

**(b)** Bar chart showing the change of cholesteryl ester contents in chondrocytes following inhibition of p-Smad3 by SIS3 in the presence of TGF-β2, measured by Beyotime Amplex Red Cholesterol and Cholesteryl Ester Assay Kit. Data from three independent experiments (n = 3) were analyzed by one-way ANOVA and are presented as mean ± SD.

**Figure S18**

**Figure S18. Inhibition of TβRI decreases the expression of p-smad3 in a collective of chondrocytes in the presence of TGF‐β2.**

Representative immunofluorescence micrographs showing that blockade of TβRI by SB525334 decreased TGF-β2-induced p-Smad3 expression, especially in nuclei of a collective of chondrocytes. p-Smad3 is labeled in red (Alexa Fluor® 647), F-actin cytoskeleton is highlighted with FITC-phalloidin (green), and nuclei are stained with Dapi (blue). Images are representative of five independent experiments (n = 5). Scale bars: 50 μm (first four columns) and 15 μm (last two columns, enlarged images).

**Figure S19**

**Figure S19.** **Metabolomics reveals changes in lipid and non-lipid metabolites in TGF-β2-induced chondrocytes.**

**(a)** Bar chart showing changes in four major metabolite categories in chondrocytes induced by TGF-β2. The categories include: (1) amino acid and its metabolites (gray); (2) FA, organic acid and its derivatives (yellow); (3) nucleotide and its metabolites (green); and (4) carbohydrate and its metabolites (cyan). Fold change ≥ 2.0 & *p* < 0.05.

**(b)** Metabolite interaction analysis showing that all metabolites can form an integrated network. In addition to the 91 metabolites from four major metabolite categories, two additional compounds are included: the coenzyme and vitamin all-trans-13,14-dihydroretinol (C_20_H_32_O) and the alcohol and amine calcitriol (C_27_H_44_O_3_).

**(c)** Pie chart showing the proportion of four major metabolite categories. Amino acid metabolism (amino acid and its metabolites) accounts for 71.7% (65 metabolites); lipid metabolism (FA, organic acid and its derivatives) accounts for 15.3% (14 metabolites); nucleotide metabolism (nucleotide and its metabolites) accounts for 7.6% (7 metabolites); and carbohydrate metabolism (carbohydrate and its metabolites) accounts for 5.4% (5 metabolites).

**(d)** Pheatmaps showing metabolite abundance across four major categories. Samples 1 and 1’, 2 and 2’, 3 and 3’, 4 and 4’ are derived from the same mother cells. Fold change ≥ 2.0 & *p* < 0.05.

**(e)** Violin plot of the top 50 metabolites, including 37 amino acid metabolites (top five shown, upper), 8 lipid metabolites (shown in Figure 9e), 4 nucleotide metabolites, and 1 carbohydrate metabolite (lower).

**(f)** Violin plot showing two additional upregulated metabolites in chondrocytes induced by TGF-β2: the coenzyme and vitamin all-trans-13,14-dihydroretinol (C_20_H_32_O) and the alcohol and amine calcitriol (C_27_H_44_O_3_).

**Supplementary videos**

**Video S1-Control**

Label-free real-time cell monitoring system recording the dynamics of lipid droplets in normal chondrocytes within 1 h.

**Video S2-TGFbeta2**

Label-free real-time cell monitoring system recording the dynamics of lipid droplets in chondrocytes induced by TGF-β2 at 5 ng/ml within 1 h.
